# Pervasive Variation of Transcription Factor Orthologs Contributes to Regulatory Network Evolution

**DOI:** 10.1371/journal.pgen.1005011

**Published:** 2015-03-06

**Authors:** Shilpa Nadimpalli, Anton V. Persikov, Mona Singh

**Affiliations:** 1 Department of Computer Science, Princeton University, Princeton, New Jersey, United States of America; 2 Lewis-Sigler Institute for Integrative Genomics, Princeton University, Princeton, New Jersey, United States of America; University of Chicago, UNITED STATES

## Abstract

Differences in transcriptional regulatory networks underlie much of the phenotypic variation observed across organisms. Changes to cis-regulatory elements are widely believed to be the predominant means by which regulatory networks evolve, yet examples of regulatory network divergence due to transcription factor (TF) variation have also been observed. To systematically ascertain the extent to which TFs contribute to regulatory divergence, we analyzed the evolution of the largest class of metazoan TFs, Cys2-His2 zinc finger (C2H2-ZF) TFs, across 12 *Drosophila* species spanning ~45 million years of evolution. Remarkably, we uncovered that a significant fraction of all C2H2-ZF 1-to-1 orthologs in flies exhibit variations that can affect their DNA-binding specificities. In addition to loss and recruitment of C2H2-ZF domains, we found diverging DNA-contacting residues in ~44% of domains shared between *D. melanogaster* and the other fly species. These diverging DNA-contacting residues, found in ~70% of the *D. melanogaster* C2H2-ZF genes in our analysis and corresponding to ~26% of all annotated *D. melanogaster* TFs, show evidence of functional constraint: they tend to be conserved across phylogenetic clades and evolve slower than other diverging residues. These same variations were rarely found as polymorphisms within a population of *D. melanogaster* flies, indicating their rapid fixation. The predicted specificities of these dynamic domains gradually change across phylogenetic distances, suggesting stepwise evolutionary trajectories for TF divergence. Further, whereas proteins with conserved C2H2-ZF domains are enriched in developmental functions, those with varying domains exhibit no functional enrichments. Our work suggests that a subset of highly dynamic and largely unstudied TFs are a likely source of regulatory variation in *Drosophila* and other metazoans.

## Author Summary

The phenotypic differences observed between closely related organisms are thought to be due largely to changes in regulatory networks. Changes in transcriptional networks can occur via mutations in *cis* binding sites, for which there are numerous known examples, as well as via binding specificity variation in transcription factors (TFs), a less studied phenomenon that has been observed primarily in multi-gene families. Though large-scale experimental studies ascertaining the extent to which TFs contribute to regulatory network variation across organisms are lacking and would be time-consuming, computational methods can begin to address this challenge. Here, we present a systematic, large-scale analysis of DNA-binding specificity evolution in TF orthologs by computationally leveraging specific features of Cys_2_-His_2_ zinc finger proteins, the largest class of TFs in animals and major components of their regulatory programs. We find not only that divergence of DNA-binding residues in 1-to-1 orthologous C2H2-ZFs is pervasive but also that these changes show evidence of functional constraint and occur in a gradual, evolutionarily viable manner. We conclude that the diversification of orthologous TFs has most likely played a major and largely unstudied role in gene regulatory network evolution in metazoans.

## Introduction

Differences in regulatory networks have been proposed to be one of the major determinants of the phenotypic variations observed across organisms [1]. There are two ways by which regulatory networks evolve: changes in *cis* or *trans*. The predominant view is that regulatory evolution results mainly from the gain and loss of binding sites in *cis*-regulatory regions because incremental, evolutionarily viable steps can occur [2–5]. Mutations in transcription factors (TFs), on the other hand, can affect the expression of multiple genes and are thought therefore to be more likely to have detrimental consequences [6–9]. Nevertheless, case studies of specific biological systems have revealed instances of regulatory divergence stemming from TF variation. These variations include gene loss as well as gene duplication where the subsequent paralogs exhibit gain and loss of effector domains, changes in interactions with other regulatory proteins, or novel TF binding potential [10–15]. Specific cases of variations in non-duplicated TFs are also known; an example of 1-to-1 orthologous plant TFs with differing binding specificities was recently discovered [16], along with a homeodomain TF in animals where the addition of a functionally important transcriptional repressor domain is found in insect orthologs [17, 18]. However, a large-scale experimental study ascertaining the extent to which TF variation may contribute to overall regulatory network evolution is still lacking; it would require determining DNA-binding specificities or genomic occupancies for numerous TFs across a diverse set of organisms. Computational methods can begin to address this challenge by leveraging specific features of TFs.

TFs come in distinct structural classes based upon their incorporation of various DNA-binding domains. For many of these domains, the amino acids conferring DNA-binding specificity are known. This provides a platform to assess TF variation via comparative sequence analysis. The Cys_2_-His_2_ zinc finger (C2H2-ZF) TFs in particular are an excellent system to probe for variation, as C2H2-ZF domains have a conserved modular structure with binding specificity conferred largely by four DNA-contacting residues within the domain’s alpha-helix [19]. Further, they constitute the largest group of TFs in higher metazoans [20], making up nearly half of all annotated TFs in human, and are major participants in regulatory programs. A C2H2-ZF domain can specify a wide range of three or four base pair targets, and tandem arrays of these domains bind contiguous DNA sequences, giving C2H2-ZF genes the ability to recognize an incredibly diverse set of motifs [21]. These features of C2H2-ZFs allow us to make binding specificity predictions of reasonably high quality for this TF family [22–26].

Previous evolutionary analyses of C2H2-ZF genes revealed a dichotomy in conservation patterns of this family. Tandemly-duplicated C2H2-ZF paralogs exhibit differences in their C2H2-ZF and effector domain counts and can be highly dynamic across short evolutionary distances [27]. The subset of C2H2-ZF KRAB repressor regulators in particular have undergone recent, rapid expansion and divergence in primates and show evidence of adaptive evolution in their DNA-binding domains in human [13, 28–30]. However, such divergence has been found primarily in extremely recent and often species-specific expansions of C2H2-ZFs [31]. In contrast, examples of single-copy 1-to-1 orthologous C2H2-ZF genes have been shown to be highly conserved across large evolutionary distances [27, 32–35]. *Prdm9*, a C2H2-ZF gene that mediates homologous recombination but is not known to be a TF, is a notable exception to this trend, and is highly dynamic between and within species despite being single-copy [36–40]. Several other orthologous C2H2-ZF genes have been found to diverge across vertebrates, primarily through the gain and loss of C2H2-ZF domains [27, 31]. More generally, however, it is widely believed that 1-to-1 orthologous TFs tend to maintain their DNA-binding specificities whereas paralogous TFs are free to vary [41].

**Fig 1:**
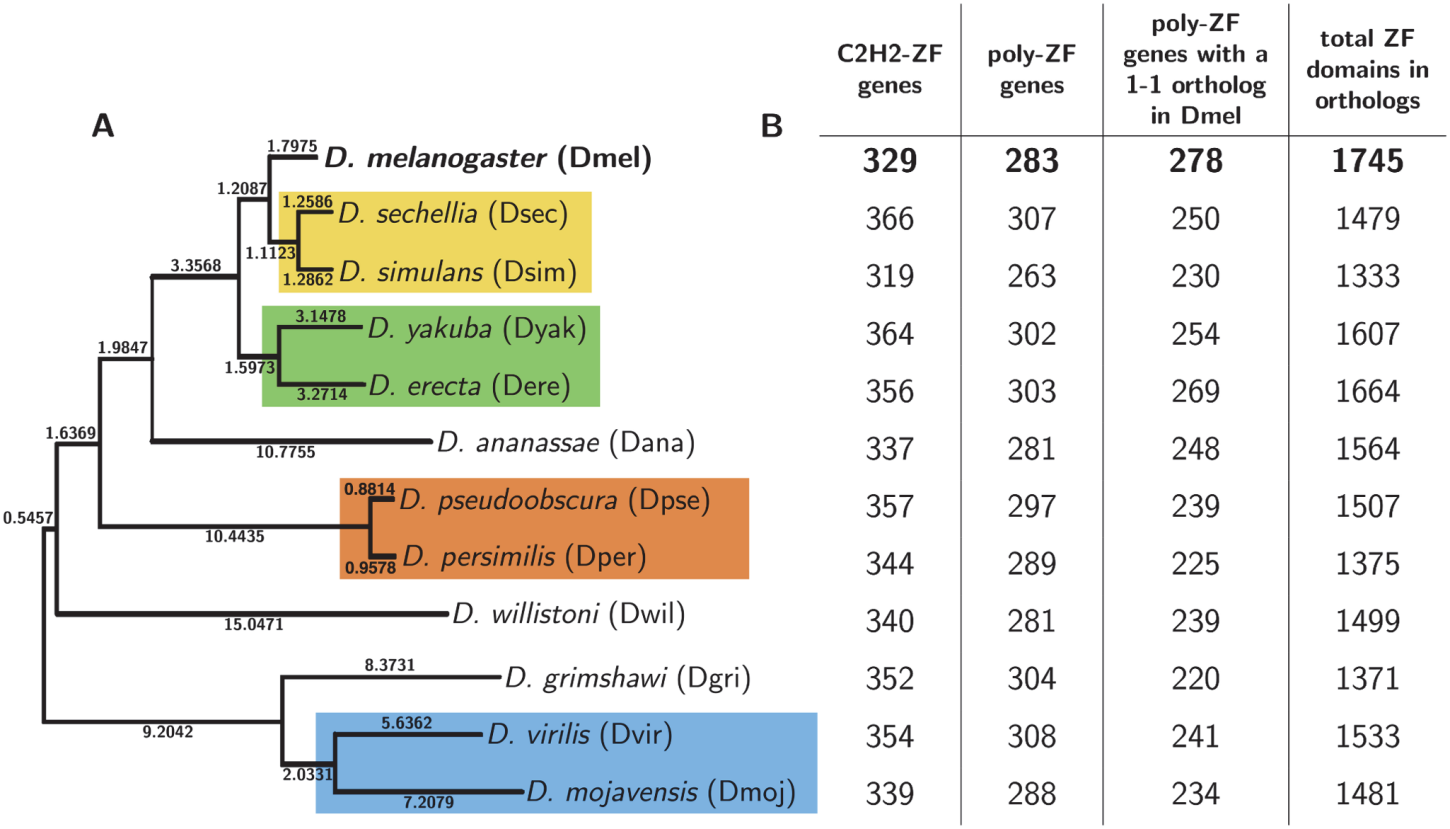
Phylogenetic tree relating 12 *Drosophila* species. (A) Phylogenetic tree of 12 *Drosophila* species. The four-letter abbreviations of the species are given with *D. melanogaster*, the reference sequence, in bold. Branch lengths are as reported in the UCSC Genome Browser. The most closely related pairs of species are highlighted in colored boxes. (B) Columns correspond to counts of, for each species: C2H2-ZF genes, C2H2-ZF genes with 2+ C2H2-ZF domains (poly-ZF genes), poly-ZF genes with a 1-to-1 ortholog in *D. melanogaster*, and the number of domains found in the set of poly-ZF genes with 1-to-1 orthologs in *D. melanogaster*. In the *D. melanogaster* row, the last two columns correspond to the count of poly-ZF genes with 1-to-1 orthologs in any of the other 11 fly species and the number of domains found in this set of poly-ZF genes.

In this paper, we analyze 1-to-1 orthologous C2H2-ZF TFs across closely related species. We leverage the well-understood binding interface of C2H2-ZFs to evaluate DNA-binding specificity changes resulting from C2H2-ZF variation. We focus on C2H2-ZFs in the 12 sequenced *Drosophila* species (phylogenetic tree in Fig. 1A), as these species benefit from relatively high-quality assembled genomes [42]. Further, as a result of their ∼45 million years of evolutionary divergence [43], they exhibit extensive regulatory variation [44, 45] and diversity in terms of morphology, physiology and ecology [46]. The flies are an ideal model organism set for our study because they have several hundred C2H2-ZF genes which are found in well-established orthologous relationships. This is in contrast to primate genomes where large-scale species-specific expansions complicate 1-to-1 orthology determination.

To assess change, we consider only C2H2-ZF genes that are in 1-to-1 orthologous relationships between *D. melanogaster*, which we use as a reference species, and each of the 11 other fly species. We find evidence of functional modifications to DNA-binding potential in a significant proportion of these genes. Furthermore, these changes often result in increasingly diverse predicted DNA-recognition motifs as evolutionary distance from *D. melanogaster* increases, implying that C2H2-ZF DNA-binding specificities may evolve gradually in evolutionarily viable steps. Our findings challenge the assumption that 1-to-1 orthologous TFs are always highly conserved and provide evidence that binding specificity modifications in single-copy TFs may play an important role in the regulatory evolution of *Drosophila* and other higher metazoans.

## Results

### C2H2-ZF Domains and Orthogroup Dataset

The initial step of our framework to assess variation in C2H2-ZFs was to assemble groups of orthologs (orthogroups) of C2H2-ZF genes across the 12 fly species (Fig. 1A). We identified all C2H2-ZF domains and sequences in these species using Pfam [47] and HMMER [48] and determined 1-to-1 orthogroups from existing Flybase [43] annotations. We then augmented this set using the UCSC Genome Browser [49] whole genome fly alignment, resulting in a dataset of all C2H2-ZF sequences in the *Drosophila* species (Methods M1–M3).

C2H2-ZF domains are known to primarily work in tandem to specify DNA motifs [50] (S1B Fig.), and so we include only those C2H2-ZF genes with 2+ C2H2-ZF domains in our analysis; we refer to these genes as poly-ZF. Tandem C2H2-ZF domains that are separated by canonical linkers—stretches of 5 to 12 amino acids, most often matching the expression TGE[K|R]P[F|Y]X (S1C Fig.)—have the strongest structural evidence for DNA binding [19, 21]. We refer to all domains that are bordered by at least one canonical linker, as defined above, to be “canonically linked.” In *D. melanogaster*, of the 329 genes with at least one C2H2-ZF domain, 283 have multiple C2H2-ZF domains, and 246 of those contain canonically linked domains.

We found from 319 to 366 genes with at least one C2H2-ZF domain in each of the 12 *Drosophila* species, 263 to 308 of which were poly-ZF (Fig. 1B, cols. 1–2), in accordance with previous studies’ findings [13, 21]. We found 278 (98.2%) poly-ZF genes in *D. melanogaster* with a 1-to-1 ortholog in at least one other fly species, and 165 (59.4%) of these were in 1-to-1 relationships across all species. These 278 1-to-1 orthologous poly-ZFs constitute 36.9% of the estimated 753 TFs in *D. melanogaster* [51]. In each non-*melanogaster* species, 72.4% to 88.8% of poly-ZF genes had a 1-to-1 ortholog in *D. melanogaster* (Fig. 1B, col. 3). In the non-*melanogaster* poly-ZF genes with 1-to-1 orthologs in *D. melanogaster*, we identified 1000+ C2H2-ZF domains per species (Fig. 1B, col. 4) that are used for comparative analysis in further steps of our framework.

### Substantial Loss and Recruitment of C2H2-ZF Domains Relative to *D. melanogaster*

We first assessed the loss and gain of C2H2-ZF domains across our orthogroups, as the number and arrangement of C2H2-ZF domains likely affects the binding specificity of each poly-ZF gene. *D. melanogaster* domains are considered “lost” in each non-*melanogaster* species without a corresponding aligned domain; *D. melanogaster* domains with no aligned domains in any of the other fly species are ignored because they most likely are species-specific *D. melanogaster* gains. Conversely, domains from non-*melanogaster* sequences that did not align back to a *D. melanogaster* domain are considered “gains” with respect to the reference.

**Fig 2:**
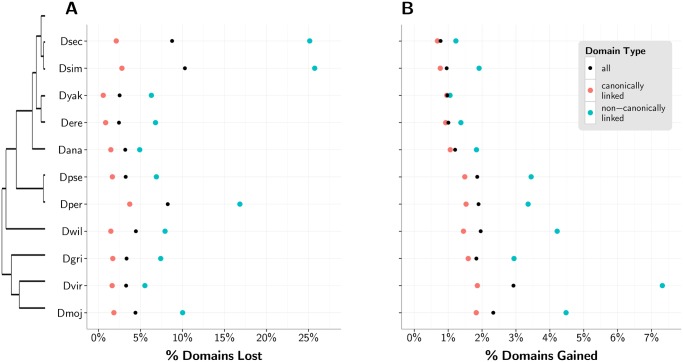
Loss and recruitment of C2H2-ZF domains with respect to *D. melanogaster* reference. (A) Percent of *D. melanogaster* domains lost in each non-reference species for all domains (black) and separately for non-canonically linked (blue) and canonically linked (red) domains, with a phylogenetic tree relating the fly species to the left. (B) Percent of domains gained by each non-*melanogaster* species.

The loss and gain of C2H2-ZF domains was recently identified as the major source of divergence in vertebrate ZF paralogs and orthologs [31]. We quantify this phenomenon in 1-to-1 orthologous TFs in *D. melanogaster*, where we find that between 2.4% and 10.3% of domains were lost in the other fly species (Fig. 2A), and between 0.8% and 2.9% of domains from non-*melanogaster* species were gained with respect to the reference (Fig. 2B). A notable 24.8% of all non-reference poly-ZF genes in 1-to-1 orthologous relationships with a *D. melanogaster* gene have lost or gained a C2H2-ZF domain with respect to the reference. 75.6% of gains or losses occur outside of or at an end of an array of canonically linked domains. The proportion of domains lost and gained in the non-*melanogaster* species with respect to the reference increases as the phylogenetic distance from *D. melanogaster* increases. When considering only canonically linked C2H2-ZF domains, we see the same overall phylogenetic trends, albeit at a lower level.

We note that *D. melanogaster* benefits from more complete sequencing coverage in comparison to the the other fly genomes [42], and relatively poor coverage and subsequent inaccurate sequence assembly would result in a greater number of unidentified or misidentified domains in those genomes. *D. sechellia, D. simulans*, and *D. persimilis*, which exhibit the greatest relative C2H2-ZF domain loss (Fig. 2A), also have the lowest relative coverage: 4.9x, 2.1x, and 4.1x, respectively, compared to between 8.4x and 11.0x for the other species. For this reason, the C2H2-ZF domain gains relative to *D. melanogaster* are especially noteworthy, while some of the apparent domain losses, especially from *D. sechellia, D. simulans*, and *D. persimilis*, may be due to incomplete assemblies.

### Pervasive Variation in Specificity-Determining Residues in Aligned C2H2-ZF Domains

**Fig 3:**
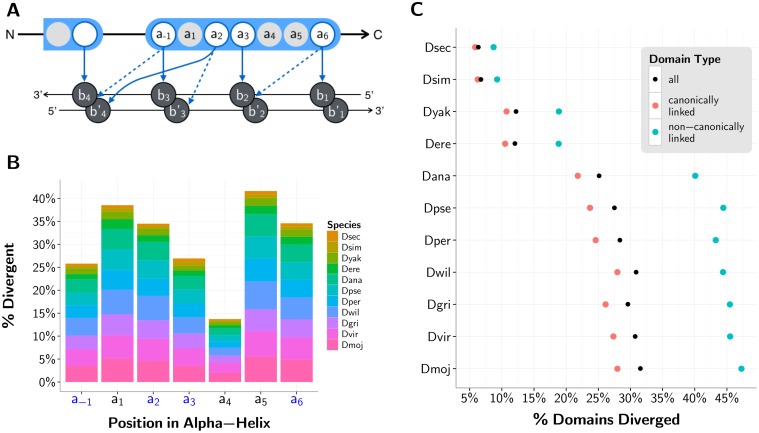
C2H2-ZF domain divergence with respect to *D. melanogaster* reference. (A) Schematic of a C2H2-ZF protein—DNA interface under the 7-contact model [53]. Amino acids within the depicted finger are numbered according to their relative position from the start of the alpha helix within the C2H2-ZF domain, with *a_-1_* indicating the position before the start of the helix. Bases *b_1_, b_2_, b_3_* and *b_4_* are numbered sequentially from 5’ to 3’ of the primary DNA strand; the complementary bases are denoted by *b_1_’, b_2_’, b_3_’* and *b_4_’*. Contacts between amino acids and bases are shown in arrows, with four specificity-determining amino acids *a_-1_, a_2_, a_3_* and *a_6_* making these contacts. Solid arrows depict the four “canonical” contacts between ZF domains and DNA [52], and dashed arrows depict three additional contacts that are used in our predictions of binding specificity [53]. (B) Histogram showing the percent divergence per species by position within the C2H2-ZF domain’s alpha-helix (-1 to 6) for all canonically linked domains. The columns with blue labels in the *x*-axis correspond to positions that interact with DNA in the 7-contact model. (C) Percent of all (black), canonically linked (blue) and non-canonically linked (red) aligned domains in each non-reference fly species with a divergent residue (as compared to the *D. melanogaster* reference) in positions -1, 2, 3, and/or 6.

Binding specificity may also be altered as a result of deviations in the DNA-contacting, specificity conferring residues in positions -1, 2, 3, or 6 of the C2H2-ZF domain [52] (Fig. 3A). As expected, with the exception of structurally constrained position 4, these four functional sites are more conserved than the neighboring, non-DNA-contacting residues within the domain’s alpha-helix. However, these functional sites still show substantial divergence (Fig. 3B). We consider an aligned domain in any non-reference fly species to be “diverged” if at least one of its residues from positions -1, 2, 3, or 6 has diverged from the *D. melanogaster* reference. Of the > 98% of domains from poly-ZF genes that aligned between the non-reference sequences and their orthologs in *D. melanogaster*, we observe from 6.3% of domains diverged (in *D. sechellia*, last common ancester [LCA] with *D. melanogaster* ∼2 Mya) to a substantial 31.5% of domains diverged (in *D. mojavensis*, LCA with *D. melanogaster* ∼45 Mya) (Fig. 3C). These divergent domains are not confined to a small subset of genes: across the 11 non-reference fly species, 19.5% to 62.4% of poly-ZF genes with 1-to-1 orthologs in *D. melanogaster* contain at least one divergent C2H2-ZF domain. Moreover, as with the proportion of domains lost and gained with respect to the reference, the proportion of domains diverged steadily increases as phylogenetic distance from *D. melanogaster* increases. The same trend with slightly lower overall divergence is observed in the subset of canonically linked domains. Of the 37.6% of domains situated in the middle of canonically linked arrays, 15.3% contain divergent binding residues. Of the remaining domains outside of or flanking canonically linked arrays, 25.1% contain divergent binding residues. Arrays of canonically linked domains appear to be under stricter constraints than singleton domains are (S2A-C Fig.). Altogether, changes in these DNA-contacting residues are substantially more frequent than the complete gain or loss of C2H2-ZF domains.

### Functional and Evolutionary Importance of Divergent Sites

#### Diverging DNA-binding residues show conservation within phylogenetic clades

**Fig 4:**
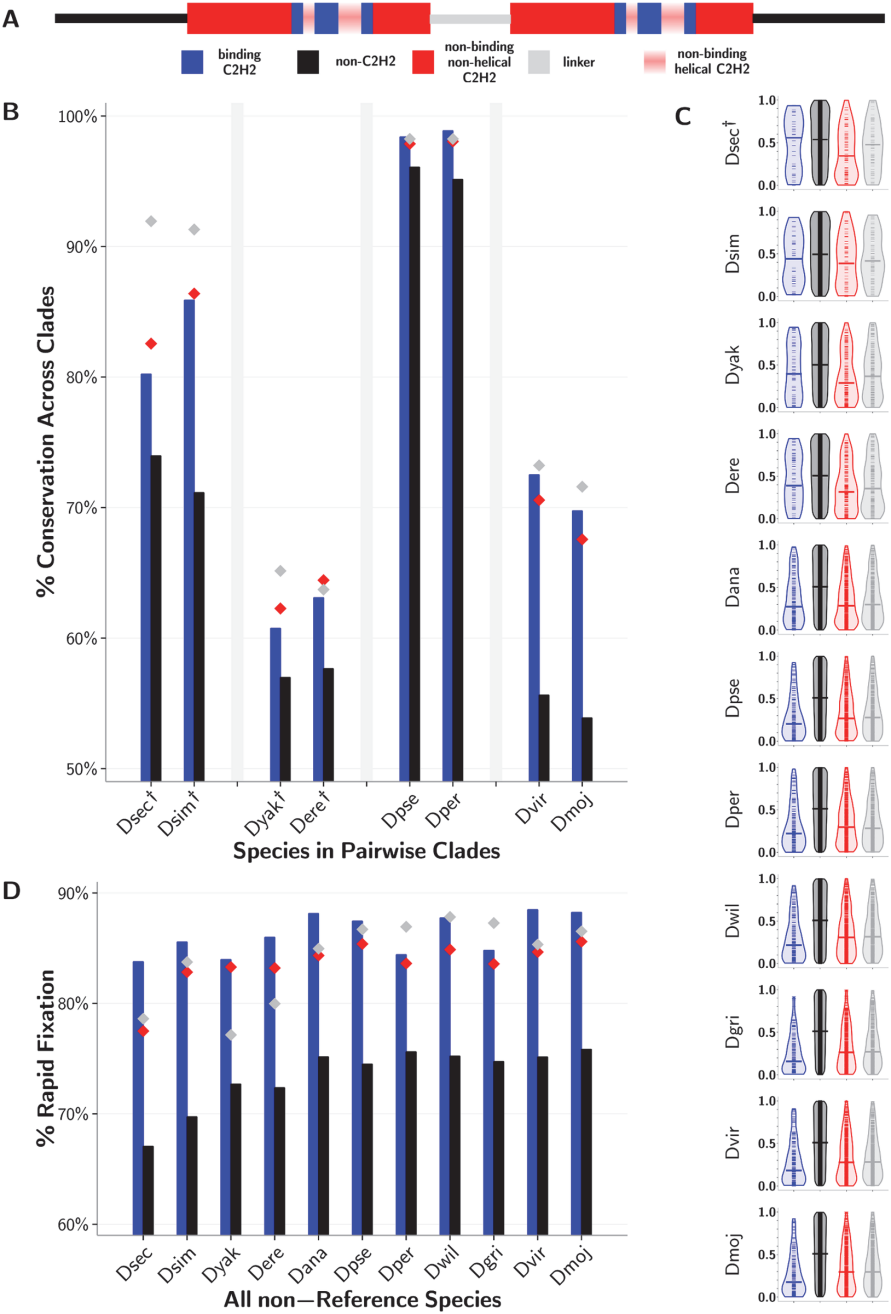
Functional importance of C2H2-ZF gene residues. (A) Legend depicting a sequence with non-C2H2-ZF domain residues (black), residues in non-binding regions of the C2H2-ZF domain outside of the alpha-helix (red), the four DNA-binding residues in the alpha-helix (blue), and linker regions between adjacent canonically linked C2H2-ZF domains (gray). Positions 1, 4, and 5 in the alpha-helix (pink) are not included in the analysis because while they are typically not DNA binding, they are found in the recognition helix. (B) Percent of divergent residues for each of the four previously described residue classes with a matching mutation in the most closely paired species. Closely-related pairs of species are grouped on the *x*-axis. Residues corresponding to DNA-contacting residues (blue) and background residues (black) are represented as bars for visual purposes only, emphasizing the trend that DNA-contacting residues are more often conserved across clades than are background residues. Corresponding values for linker (gray) and non-binding C2H2-ZF (red) residues are represented as colored diamonds. Differences between the DNA-binding and background residues that are not significant at the *p* < 0.001 level using a binomial test are marked by daggers (†). (C) Ranks of divergent residues based on evolutionary rate as predicted by Rate4Site [55] that have been 0-to-1 normalized. These plots are violin plots, where the dynamic widths of the violins correspond to the relative density of points in the distribution, and the medians are given by horizontal lines. The areas of the violins are equal per plot. Differences between the binding and background residues are calculated using a Wilcoxon test, and those differences that are not significant at the *p* < 0.001 level are marked by daggers. (D) Percent of residues found to diverge between *D. melanogaster* and each other fly species that did *not* correspond to a polymorphic site in *D. melanogaster* population data. Values for different residue types as well as significance between DNA-binding and background residues as calculated using a binomial test are both represented as in panel B. Exact *p*-values for all species, ranging from approximately 0.2 to 1e-22 (Part A), 0.02 to 1e-56 (Part B) and 1e-16 to 1e-165 (Part C) can be found in S1 Table.

We reasoned that changes in these single-copy TFs relative to *D. melanogaster* that are functionally important are likely to be conserved across phylogenetic clades. To test this, we extracted sequences from the most closely related pairs of species—*D. sechellia* and *D. simulans* (LCA < 2 Mya), *D. pseudoobscura* and *D. persimilis* (LCA ∼2 Mya), *D. yakuba* and *D. erecta* (LCA ∼5 Mya), and *D. virilis* and *D. mojavensis* (LCA ∼25 Mya)—and asked how often a particular mutation with respect to the reference in one species was supported by an identical mutation in its partner species. In all cases, divergent DNA-binding residues in poly-ZF genes from each non-*melanogaster* fly species exhibit clade support more often than background divergent residues in these genes (Fig. 4A-B); these changes are significant (*p* < 0.001, binomial test) in 4 species, with small sample sizes a limiting factor in the other species (S1 Table). Residues within and between adjacent C2H2-ZFs are also under structural constraints and may be implicated in secondary binding specificities [54], resulting in their high conservation according to the clade support measure. This trend is particularly apparent in the species closest to *D. melanogaster* with fewer overall divergent residues. Altogether, this analysis suggests that the substantial binding residue variation we see across species is functional rather than random.

#### Relatively low evolutionary rate of diverging DNA-binding residues

To further support the claim that observed variations in C2H2-ZF binding residues are functionally important, we estimate site-based evolutionary rates using Rate4Site [55] for all divergent residues per sequence per orthogroup (Fig. 4C). For each sequence, we ranked its divergent residues from lowest to highest evolutionary rates, and normalized these ranks to values between 0 and 1. In each non-reference species across all orthogroups, divergent binding residues evolve more slowly than background residues outside of C2H2-ZF domains (*p* < 0.001 in the 10 species furthest from *D. melanogaster*, Wilcoxon test; S1 Table). Because we consider only divergent residues in each sequence, this signal is strongest in species with a large number of total divergent residues per sequence, as normalized ranks are more continuous in these cases and therefore differences between the four classes of residues (i.e., specificity-conferring binding residues, background, non-helical C2H2 residues, and linker regions) are apparent with higher resolution. In the flies furthest away from *D. melanogaster*, where the most variation from *D. melanogaster* is observed, the divergent binding residues exhibit the slowest evolutionary rate relative to all other classes of divergent residues, including the structurally constrained non-binding regions within domains and the linker regions between domains in poly-ZF genes. In the four species closest to *D. melanogaster*, non-binding residues in C2H2-ZF domains appear to evolve as slowly as binding residues themselves, as the low number of total divergent residues per sequence (S1 Table) restricts the resolution of differences between these residue classes.

#### Population analysis suggests positive selection in evolutionary history

We have shown that divergent binding residues are under functional constraints, yet the pervasiveness of such changes in these 1-to-1 orthologs suggests these deviations may confer an evolutionary advantage and were selected for when they arose. The classic approach for detecting positive selection from cross-species sequences is to calculate dN/dS, the ratio of observed nonsynonymous mutations over all possible nonsynonymously mutable sites to observed synonymous mutations over all possible synonymously mutable sites [56]. However, we found that across the *Drosophila* species, dS naturally saturates and thus impedes a positive selection signal of dN/dS > 1, suggesting that this measure is inappropriate for use across the evolutionary distances considered here [57].

In order to detect positive selection in the evolutionarily dispersed fly species, therefore, we utilize an alternate approach which considers both cross-species sequences and within-species population data. If a nonsynonymous mutation was neutral and accumulates via random genetic drift, it is more likely to persist as a polymorphism within a population, whereas if such a mutation was advantageous and became fixed rapidly through positive selection, finding nonsynonymous mutations in the same location in population data would be highly unlikely [58]. Consequently, for each divergent residue in each non-reference species, we asked whether that same site was or was not polymorphic in a population of 139 *D. melanogaster* organisms [59] (Fig. 4D). In every species, a greater proportion of divergent binding residues are disjoint from polymorphic sites than divergent background residues are (*p* < 1e-15 in all species, binomial test; S1 Table), and in 8 of the 11 species these proportions are greater than those for all other types of diverging residues. In four species, linker regions between domains, which may impact overall specificity by affecting flexibility of canonical binding arrays and the positioning of C2H2-ZF domains within them, had residue changes present as polymorphisms as often as the binding residues themselves. In two species, diverging non-helical residues, which may alter the structure of the DNA-binding domains, also overlapped with polymorphic sites in *D. melanogaster* as rarely as binding residues did. For the set of diverging residues in each of the 11 species separately, we also computed site frequency spectra [60] to analyze polymorphic sites (S3 Fig.). These polymorphic sites are heavily skewed towards smaller minor allele frequencies, with this trend most evident in the diverging DNA-contacting residues. Altogether, these analyses of a *D. melanogaster* population suggest that a greater proportion of binding residue divergences in each species were likely advantageous rather than neutral as compared with other variations within poly-ZF genes.

### Divergent Residues Lead to Distinct Computationally-Predicted Specificities

We next aimed to ascertain whether and how the variation we observe in poly-ZF orthologs changes binding specificity, as it is possible that distinct assignments of binding residues still specify the same overall recognition motif [26, 61]. We predicted the specificity of each C2H2-ZF domain with a predictor [24, 62] that utilizes a linear support vector machine based on an expanded structural model (Fig. 3A); this method is referred to as SVM. Since no method can predict binding specificity perfectly and consensus predictions are more likely to be correct (S1 Text, S2 Table), we compared the SVM predictions to those produced by an independent predictor referred to as ML that uses a probabilistic recognition code generated via maximum likelihood [22], and a random forest based predictor referred to as RF [25]. We calculate the average Pearson correlation coefficients (PCCs) across positions b1 through b4 between SVM predicted position weight matrices (PWMs) and ML and RF PWMs, and consider only the subset of SVM predictions with average PCCs > 0.25 to either of the corresponding ML or RF predictions (S4 Fig.). Of the 17734 aligned binding domains from all 12 fly species, 87.3% passed this confidence threshold; thus, overall there is good agreement between the independent methods on predicted DNA-binding specificities. Results using alternate confidence thresholds of PCC > 0.0, PCC > 0.5 and PCC > 0.75 are found in S3 Table.

We compared the SVM-predicted PWM for each divergent domain in a non-*melanogaster* species to the predicted PWM for the corresponding, aligned domain in its *D. melanogaster* ortholog by calculating the average PCC across positions b1 through b4. Overall, 74.2% of divergent domains over the 11 flies exhibit a PCC < 0.25 from their reference domain in at least one predicted position (S5A Fig.). In six non-reference fly species, 100% of all divergent domains exhibit a PCC < 1 from their reference domains in at least one predicted position. Of the remaining five species, < 1% of divergent domains do not show a significant change in predicted specificity in any position compared to their aligned *D. melanogaster* reference domains. Many domains from non-*melanogaster* species exhibit a diverged specificity from the reference in more than one predicted position (S5B Fig.). Overall, this analysis suggests that the divergent binding residues within C2H2-ZF domains likely result in changed DNA-binding specificities.

#### Predicted binding specificities change gradually over evolutionary distance

**Fig 5:**
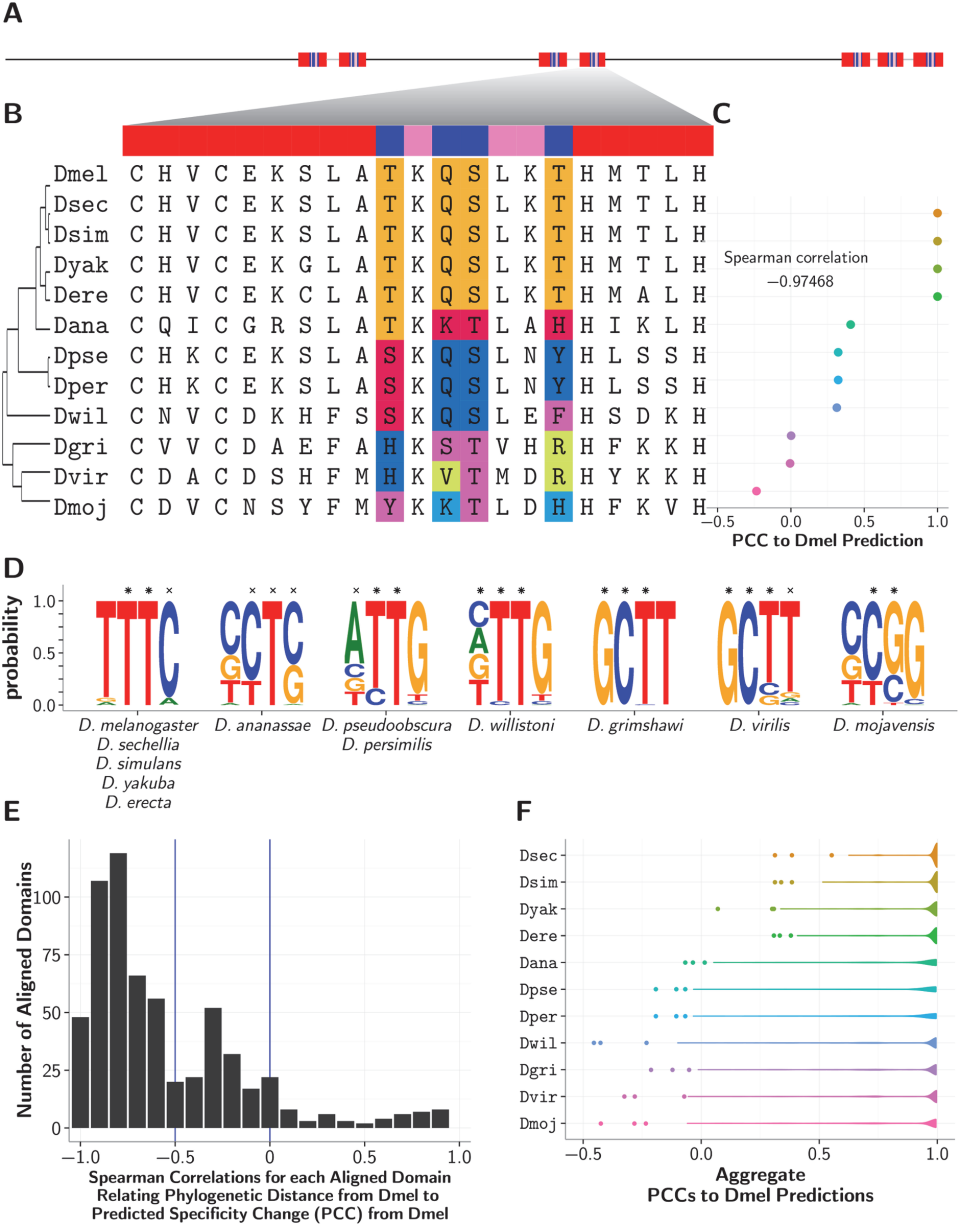
Example of a varying *D. melanogaster* C2H2-ZF domain. (A) Layout of the seven C2H2-ZF domains in *D. melanogaster* protein FBpp0072605. All domains are found in three canonically linked arrays of sizes 2, 2, and 3 respectively. Both domains in the middle array and domains 2 and 5 located at the end of the first array and start of the last array also exhibit divergent binding residues. (B) Closeup of the 4th domain in the protein, with phylogenetic tree and multiple alignment of the aligned domains from the other fly species. (C) Average (across positions b1–b4) Pearson correlation coefficients (PCCs) between non-reference and *D. melanogaster* SVM predicted specificities by species. The Spearman correlation, relating non-*melanogaster* predicted specificity change to phylogenetic distance from reference *D. melanogaster*, is also shown and implies that specificity changes increase gradually with distance from the reference. (D) Frequency plots of the PWMs generated by WebLogo [63] representing unique binding specificities, predicted by the SVM method, ordered by phylogenetic distance from *D. melanogaster*, and labeled with the species whose domains had that corresponding binding specificity. Predicted positions with a PCC > 0.25 to one of either the ML or RF corresponding predictions are marked with a ×, and positions with a PCC > 0.25 to both the ML and RF corresponding predictions are marked with a *. (E) Distribution of Spearman correlations for each aligned domain (as in Part C) relating non-*melanogaster* predicted specificity change to phylogenetic distance from reference *D. melanogaster*. (F) Violin plots depicting the distributions of PCCs between predicted specificities for non-reference domains and their aligned domains in *D. melanogaster* orthologs.

To establish how specificity may change in relation to phylogenetic distance, we compared the predicted PWM for each *D. melanogaster* domain to those PWMs predicted for every corresponding aligned domain from the other flies (example domain multiple alignment in Fig. 5A-B). In the majority of such domain alignments between *D. melanogaster* and the other fly species, we note that as the phylogenetic (species) distance from *D. melanogaster* increases, the corresponding domain’s predicted specificity change from *D. melanogaster* also tends to increase. Specifically, we measure change between *D. melanogaster* and non-*melanogaster* predicted specificities using PCC, where a lower PCC implies greater change, and so we see that an increase in phylogenetic distance is correlated with a decrease in PCC (Fig. 5C-E). As such, Spearman correlations relating phylogenetic distance to change in predicted specificity (as measured by PCC) were < 0 for 88.7% of domain alignments and < −0.5 for 65.2% of domain alignments (see S3 Table). When we group divergent non-*melanogaster* domains by species rather than by orthology to a particular *D. melanogaster* domain, we still observe this same trend, where an increase in phylogenetic distance between *D. melanogaster* and non-reference species correlates with a decrease in the PCCs between predicted *D. melanogaster* specificities and non-reference predicted specificities (Fig. 5F). Overall, our analysis of predicted specificities is consistent with a model where DNA-binding specificities diverge gradually over evolutionary time in non-duplicated, 1-to-1 poly-ZF orthologs.

### Binding Landscape Divergences Across Species

We next set out to determine whether the variation we observe in poly-ZF DNA-binding residues may result in changes in regulatory network topology. To experimentally test this, we would need experimentally-determined binding specificities and/or genomic occupancies for many poly-ZF genes across the fly species. Although we do not have TF binding data for non-*melanogaster* flies, there are poly-ZF TFs for which binding specificities or genomic binding locations have been experimentally determined in *D. melanogaster*.

We first sought to use chromatin-immunoprecipitation (ChIP) data. Of the 12 *D. melanogaster* poly-ZFs with associated ChIP data from modENCODE [64], five poly-ZFs—three of which exhibit divergences in their DNA-contacting residues and two of which are completely conserved—did not have associated PWMs representing their binding specificities available in the Fly Factor Survey [65], JASPAR [66], or public Transfac [67] databases, thereby precluding any efforts to determine whether these TFs bind in the other fly genomes. The remaining seven poly-ZFs are conserved TFs involved in development; thus, we would not be able to compare how the diverged and conserved poly-ZF genes in this set differ with respect to the loss of binding sites in the non-reference fly genomes. Because most ChIP studies have been carried out at various developmental stages in *D. melanogaster* and because, as we show in the next section, conserved poly-ZFs are enriched for developmental functions whereas diverged poly-ZFs are not, it is not surprising that few divergent poly-ZFs have associated ChIP data or specific binding at these developmental stages.

**Fig 6:**
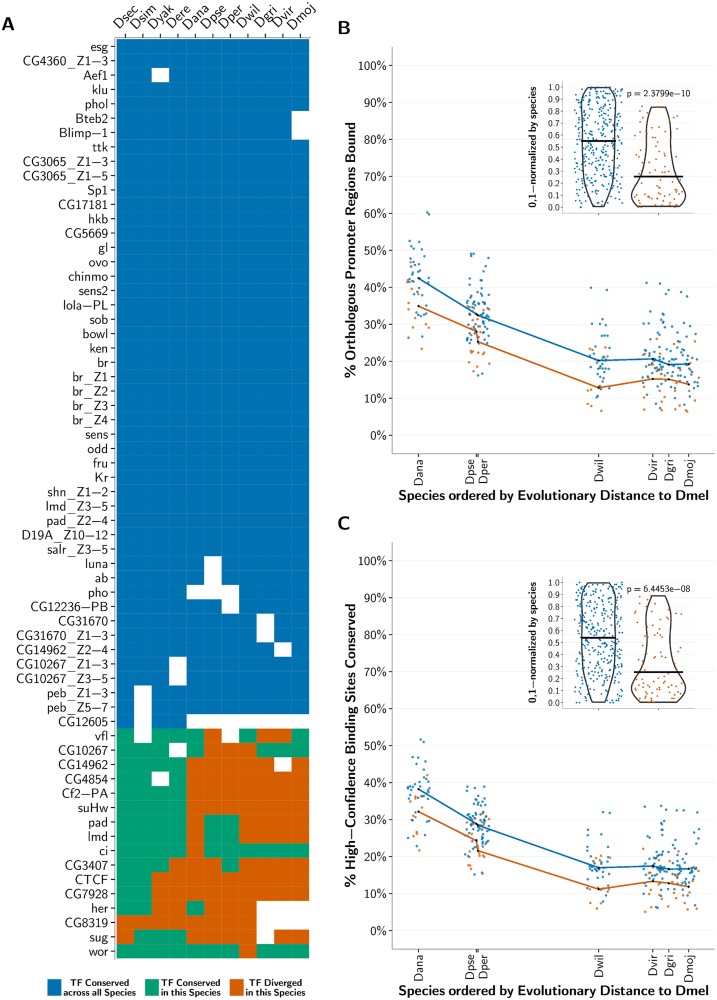
Conservation of predicted binding motifs for experimentally derived PWMs across species. (A) The list of analyzed experimentally determined C2H2-ZF binding specificity motifs (PWMs) within *D. melanogaster* along with a heat map representing the conservation of the corresponding protein construct across the fly species; note that each PWM was determined either for an entire protein or just a fragment of it. In the heat map, white depicts that a 1-to-1 ortholog for the corresponding C2H2-ZF protein in *D. melanogaster* was not present in that species; blue depicts that the DNA-contacting residues within the C2H2-ZF construct are conserved across all the flies; green depicts that the DNA-contacting residues within the C2H2-ZF construct did not diverge in that species, but one or more of these residues diverged in one or more orthologs in the other fly species; and orange depicts that the C2H2-ZF in the current species diverged from its 1-to-1 ortholog in *D. melanogaster* in at least one DNA-contacting residue within the protein construct. (B) For each species, ordered on the *x*-axis by its relative evolutionary distance from *D. melanogaster*, we plot for each PWM in panel A the fraction of promoters predicted to be bound in *D. melanogaster* whose orthologous regions within the species are also predicted to be bound. Blue points correspond to C2H2-ZFs conserved across all the flies, and orange points correspond to C2H2-ZFs that diverge in the current species. The medians of the conserved and diverged C2H2-ZFs for each species are computed and plotted as black points. Lines connecting these median points are drawn for visual effect only. For each species, conserved C2H2-ZF proteins tend to bind a higher proportion of promoter regions that are orthologous to those bound in *D. melanogaster* than do diverged C2H2-ZF proteins. (Part B Inset) Violin plots showing the per-species 0, 1-normalized ranks of percent orthologous promoter regions bound, such that the rank of the lowest percentage per species maps to 0, and the rank of the highest percentage maps to 1. The *p*-value comparing the normalized percentages between conserved and diverged C2H2-ZF orthologs is calculated using a Wilcoxon test. (C) Same as part B, where *y*-axis values correspond to the percent of high-confidence *D. melanogaster* binding sites conserved in each species for each PWM. For each species, conserved C2H2-ZF proteins tend to have a higher fraction of binding sites conserved from *D. melanogaster* than do diverged C2H2-ZF proteins.

We next compiled experimentally-determined binding specificities for 52 fly poly-ZF TFs from the Fly Factor Survey, JASPAR, and public Transfac databases (Fig. 6A) and computationally mapped their binding sites using fimo [68] in the 2000 base pair promoter regions upstream of known genes in *D. melanogaster*. To obtain a subset of high-confidence binding site predictions in *D. melanogaster*, we required that the sites be conserved in the four most closely related species—*D. sechellia, D. simulans, D. yakuba*, and *D. erecta*. For each TF, we next examined whether high-confidence *D. melanogaster* binding sites are lost in the remaining seven fly species, and whether orthologous promoter regions are no longer bound in these species. In each species, we compare the fraction of binding sites lost for those TFs with completely conserved DNA-contacting residues across their 1-to-1 orthologs with the fraction lost for those TFs exhibiting some divergence in their DNA-contacting residues as compared to their *D. melanogaster* orthologs (Methods M4). We note that various features of a TF (e.g., its function) influence the extent to which its binding sites and targets vary across organisms; thus, we compare the conserved and divergent groups of TFs in aggregate.

We find that single-copy poly-ZF orthologs with divergent DNA-contacting residues are significantly more associated with a loss of bound promoter regions than are completely conserved poly-ZF orthologs (*p* < 1e-9 across all species, Wilcoxon test; Fig. 6B). Changes between the sets of genes predicted to be regulated by *D. melanogaster* poly-ZFs and the sets of genes predicted to be regulated by their orthologs in other species, therefore, are more common and pronounced when those orthologs show divergences in their DNA-binding domains. When examining individual binding sites that were predicted to be bound by a given *D. melanogaster* poly-ZF gene, we find that divergent poly-ZFs are significantly more associated with a loss of binding sites than are conserved single-copy poly-ZFs (*p* < 1e-6 across all species, Wilcoxon test; Fig. 6C). We note that relaxing our criterion for making high-confidence binding site predictions in *D. melanogaster* by requiring conservation in fewer species does not substantially alter our findings at either the level of promoters or binding sites (S6 Fig.). Altogether, these results suggest that the binding landscapes of divergent poly-ZFs are more different from their *D. melanogaster* orthologs than are those of conserved poly-ZFs, and subsequently that regulatory network topologies have most likely been affected by variation in 1-to-1 orthologous poly-ZFs.

### Diverged Poly-ZFs are Functionally Varied; Conserved Poly-ZFs are Developmentally Enriched

Do divergent poly-ZF genes exhibit distinct biological functions from the set of conserved poly-ZF genes? To answer this question, we divided the genes from our analysis into two main sets: conserved and diverged. The first set contained 82 poly-ZF genes from *D. melanogaster* with completely conserved DNA-contacting residues across all its orthologs; 28 (34.1%) had orthologs in all other fly species, and 64 (78.0%) contained canonically linked domains. The second set contained 181 *D. melanogaster* poly-ZF genes with a diverged C2H2-ZF domain in 2+ orthologs; 81 (44.8%) had orthologs in all 11 other fly species, 155 (85.6%) contained canonically linked domains, and 144 (79.6%) contained a divergent canonically linked domain.

#### Divergent poly-ZFs have limited functional annotations

We ran GO Term Finder [69] on these two gene sets to find enrichment of Gene Ontology terms from the biological process, molecular function, and cellular component association categories, excluding annotations inferred from sequence models, as the presence of C2H2-ZF domains would likely have automatically inferred transcriptional regulation and DNA-binding for all poly-ZFs. Both the conserved and divergent sets are separately enriched for DNA-templated regulation of transcription, positive or negative regulation of gene expression, and regulation of RNA metabolic process and are localized in the nucleus (*p* < 0.001, Bonferroni-corrected hypergeometric test; S4 Table). Unsurprisingly, those poly-ZF genes with conserved binding specificities are also enriched for such developmental functions as segmentation, morphogenesis, and organ development. The poly-ZF genes with divergent C2H2-ZF domains, on the other hand, exhibit no additional functional enrichments, even when considering only genes with orthologs in every species, genes with canonically linked domains, or the gene set augmented with functional protein partners from STRING [70] (version 9.1, interaction scores > 0.9).

Although no functions beyond transcriptional regulation were significantly enriched across the entire set of divergent poly-ZF genes, certain genes within this set were annotated with functions such as organ development (muscle, respiratory system, axon, wing disc), dorsal/ventral pattern formation, and neurogenesis. Indeed, several known TFs are found in the set of divergent genes. For instance, hermaphrodite (*her*), a regulator required for sexual differentiation [71], has four C2H2-ZFs, the first of which has a mutation in position 2 in *D. yakuba* and *D. erecta*, the fourth of which has a mutation in position 2 in *D. pseudoobscura* and *D. persimilis*, and the second and third of which both have mutations in position 2 in *D. willistoni*. Matotopetli (*topi*), a testis-specific regulator of meiosis and terminal differentiation [72], has 11 C2H2-ZF domains in *D. melanogaster*, of which five were mutated in the six species furthest from *D. melanogaster*, four were mutated in *D. ananassae*, two were mutated in *D. yakuba*, and one was mutated in *D. sechellia* and *D. erecta*. Tiptop (*tio*), a repressor of the teashirt TF and regulator of clypeolabrum patterning [73], has five C2H2-ZF domains, the first, third, and fifth of which have diverged from the *D. melanogaster* ortholog in seven other species. Overall, however, functional analysis reveals a clear study bias toward conserved, developmentally involved TFs.

#### Co-domain presence suggests transcriptional regulation activity

Because we excluded GO terms inferred from sequence models when looking for functional enrichment, we separately analyzed the co-domains present in the complete conserved and diverged poly-ZF gene sets to get a better sense of these genes’ functions. We downloaded domain annotations from InterPro [74]. Both of these gene sets contain the regulation-related effector domain BTB/POZ, which mediates homomeric dimerization, and additional DNA-binding AT-hook and homeobox domains. Remarkably, a third of poly-ZFs in *D. melanogaster* contain the ZAD domain; this is largely an insect-specific domain, and its prevalence in fly proteins containing C2H2-ZF domains has been noted before [75–77]. While a few proteins with ZAD domains are completely conserved across the flies, nearly 90% exhibit some divergence, with 78% falling into the divergent set as defined above. Altogether they constitute ∼40% of the divergent set. The domains found uniquely in the conserved set are DZF, a nucleotidyltransferase; SET, a histone methyltransferase found predominantly in enhancer TFs; Ovo, which plays a role in germline sex determination; SANT/Myb, another DNA-binding domain; and ELM2, a domain of unknown function. Divergent C2H2-ZF genes uniquely contain several domains implicating their regulatory activity—PHD, responsible for chromatin-mediated transcriptional regulation; PWWP, ING, WD40, and bromodomain, all important for chromatin remodeling, genome stability maintenance, protein-histone association, and cell cycle progression regulation; EPL1, involved in transcriptional activation; and BESS, TRAF, and SWR1, which direct a variety of protein-protein interactions.

#### Divergent poly-ZFs are less essential and more widespread

Additional phenotypic information derived from gene knockout experiments are available via FlyBase and modENCODE [64] for 97.3% of conserved poly-ZF genes and 96.2% of divergent poly-ZF genes. Conserved poly-ZF genes are more often essential than divergent poly-ZF genes are: gene knockouts were lethal for 23.3% of conserved and only 15.8% of diverged genes. An additional 43.8% and 21.5% of conserved and divergent genes respectively had semi-lethal, recessive lethal, or larval lethal knockouts. In concurrence with the GO term enrichment, we found that 37.0% of conserved poly-ZF gene knockouts affected phenotypes in the embryonic or larval stages, whereas only 12.0% of diverged poly-ZF knockouts had a phenotypic effect during development.

To further determine where and when poly-ZF genes affect phenotype, we looked at expression locale and levels derived from FlyAtlas [78], available for 95.8% of conserved poly-ZFs and 97.5% of diverged poly-ZFs. We considered adult, larval, and germline tissues separately (S7A Fig.). Interestingly, we found that larger proportions of divergent poly-ZFs were found in each tissue than the proportions of conserved poly-ZFs. Although divergent poly-ZFs tended to be present in a larger number of distinct tissues than conserved poly-ZFs were (S7B Fig.), their expression was consistently lower than the expression of conserved poly-ZFs in corresponding tissues (S7C Fig.).

## Discussion

Previously, binding site turnover has been shown via ChIP experiments to be an essential component in regulatory network variation across closely-related organisms [79–83] and even across individuals of the same species [84, 85]. Here we present an analysis suggesting that divergence of orthologous TFs also plays a role in regulatory variation.

Over half of the single-copy, poly-ZF 1-to-1 gene orthogroups in *Drosophila* exhibit variation with respect to the number and arrangement of DNA-binding C2H2-ZF domains and the composition of specificity-conferring residues within these domains. Variations within these specificity-determining positions are known via structural studies to influence the binding specificities of the proteins in which they are found. These mutations’ conservation across phylogenetic clades, low rate of evolution, and rapid fixation as determined by their lack of overlap with population polymorphisms further demonstrate their functional importance. Additionally, predicted specificities of C2H2-ZF domains increasingly diverge as evolutionary distance from the reference *D. melanogaster* increases, offering evidence that specificity-altering *trans* changes are feasible and occur in evolutionarily viable steps even in non-duplicated orthologs.

Though C2H2-ZF binding to RNA [86] or protein [87] rather than or in addition to DNA has been observed, several lines of evidence suggest that a large fraction of the domains in our study bind DNA. We focus on only those genes with multiple C2H2-ZF domains, a requirement for specific DNA recognition. Even when we limit our analysis to canonically linked domains, which have the strongest structural evidence for DNA-binding, we observe the same overall divergence trends. Some DNA-binding C2H2-ZFs may regulate processes other than transcription; however, GO term enrichment analysis and co-domain presence suggests that many of these poly-ZFs are regulating transcription and gene expression and are likely interacting with other protein co-factors. Altogether, this suggests that a substantial set of the divergent poly-ZF genes included in our analysis are DNA-binding TFs. However, it is also possible that the likely specificity-altering mutations we see in these DNA-binding TFs may leave overall gene expression unaffected. There are cases of divergent *cis*-regulatory sequences that do not confer a change in gene expression [88–93], review by [94], as sometimes these binding site changes are accompanied by complementary TF changes [95]. Compensatory change may occur for some of the diverging poly-ZF TFs we observe. For those poly-ZFs with experimentally-derived PWMs in *D. melanogaster*, however, we see that TF orthologs across the other fly species with diverged DNA-contacting residues are associated with significantly fewer conserved binding sites and bound promoter regions than are TF orthologs with completely conserved DNA-binding domains. This suggests that the substantial *trans* variations must result in, at minimum, modulated expression changes, as multiple *cis* mutations co-occurring with and counteracting each *trans* specificity change would be extremely unlikely.

Poly-ZFs in *D. melanogaster* that diverge across the flies appear to have several notable characteristics. They tend to have limited functional annotations and are less essential than conserved poly-ZF genes. Further, they tend to be more broadly expressed, albeit at lower levels, than poly-ZF genes whose binding specificities are conserved. Intriguingly, a substantial fraction of diverging poly-ZF genes contain ZAD domains, and the vast majority of all ZAD-containing poly-ZFs diverge in their DNA-contacting residues. Uncovering the functional roles of diverging poly-ZFs, especially those containing ZAD domains, may be a particularly promising avenue for future work.

Earlier work on C2H2-ZF genes in vertebrates has established the plasticity of this class of DNA-binding domains and the potential role these genes may play in shaping species-specific regulatory networks. In particular, the human C2H2-ZF genes that contain KRAB repressor domains have been studied in depth [28, 32, 96, 97]. The KRAB C2H2-ZF family of proteins are unique to tetrapods and have undergone major species-specific segmental and tandem duplications in mammals and primates [98]. Paralogous KRAB-ZF genes residing in these clusters exhibit frequent pseudogenization, loss and gain of binding domains, and evidence of positive selection acting on the DNA-contacting residues within these domains [13, 29, 32, 97]. These findings on paralogous genes are consistent with the long-standing belief that gene duplication followed by subsequent diversification is the primary means by which otherwise conserved genes can accrue functional divergences [99]. Where attempts have been made to identify and evaluate orthologs across species containing these expansions of KRAB-ZFs, orthologs have been found to either be deeply conserved or to exhibit differences in C2H2-ZF domain count rather than in the identities of DNA-binding residues, though a few cases of variation in DNA-binding residues have been previously reported [27, 28, 31]. We note that the plasticity of domains within these expanded C2H2-ZF gene families in vertebrates does not necessarily imply that C2H2-ZF domains in other organisms will have similar properties. Indeed, we see far fewer losses and gains of domains in 1-to-1 C2H2-ZF orthologs in flies as compared to what has been observed in C2H2-ZF gene expansions in primates, and we observe a relatively higher rate of divergence in specificity-conferring residues. It remains to be seen if divergences within DNA-contacting residues are also prevalent in single-copy orthologs of other TF families.

Although prior research has recognized the possibility of TF variation occurring in multi-gene families, it has long been thought that single-copy TFs are under stringent conservation, as loss or change of function mutations in these genes could not be masked by the functional gene products of paralogs and would thus have catastrophic effects. We cannot, of course, rule out the possibility that ancient transient gene duplications and losses have complicated the detection of 1-to-1 orthologs in *Drosophila*. However, our large-scale results on 1-to-1 C2H2-ZF orthogroups in flies are consistent with a recent experimental case study of specificity divergence of a single-copy TF in plants [16]. Here, binding specificities of 1-to-1 orthologs of the plant TF LEAFY (*lfy*) were analyzed across algal, moss, and plant species, and three distinct binding preferences were found. The *lfy* ortholog in hornworts was dubbed a “promiscuous intermediate” as it recognizes all three binding motifs with various preferences. This intermediate, which is not accompanied by a definitive ancestral gene duplication event [100, 101], highlights a means by which TF binding specificity can evolve in single-copy genes. The gradual TF variation we observe may also give rise to such analogous TF intermediates.

In conclusion, we propose that variation in 1-to-1 orthologous TFs can shape regulatory network evolution. Changes in TFs need not be catastrophic. Rather, single amino acid mutations in DNA-contacting positions may result in overall TF binding of similar targets with varying affinities. Such variations provide the opportunity for gradual evolution of binding specificity. We propose that these changes in single-copy TFs may be substantial contributors to overall regulatory evolution in *Drosophila* and in other metazoans in general.

## Materials and Methods

### M1. Sequence Collection

Translated protein sequences for the 12 sequenced fly species—*D. melanogaster* (build r6.01), *D. sechellia* (r1.3), *D. simulans* (r1.4), *D. yakuba* (r1.3), *D. erecta* (r1.3), *D. ananassae* (r1.3), *D. pseudoobscura* (r3.2), *D. persimilis* (r1.3), *D. willistoni* (r1.3), *D. mojavensis* (r1.3), *D. virillis* (r1.2), and *D. grimshawi* (r1.3)—were downloaded from FlyBase [43], version FB2014_04. Additional *D. simulans* sequences were downloaded from the Andolfatto Lab site [102]. To identify C2H2-ZF genes, HMMER’s hmmsearch (versions 2.3.2 [48] and 3.0 [103]) was run on each translated protein file using 12 Pfam HMMs [47], which were selected based upon their similarity to and presence in the same clan as the consensus C2H2-ZF profile (S1B Fig.), zf-C2H2 (PF00096)—zf-C2H2 (PF00096), zf-C2H2_2 (PF12756), zf-C2H2_6 (PF13912), zf-C2H2_jaz (PF12171), zf-C2HC_2 (PF13913), zf-H2C2_5 (PF13909), zf-met (PF12874), zf-met2 (PF12907), zf-BED (PF02892), zf-U1 (PF06220), GAGA (PF09237), DUF3449 (PF11931). Any protein sequence containing at least one HMMER hit with a bit score above the specified gathering domain threshold for that HMM was considered.

C2H2-ZF domains themselves were identified from these proteins as any HMMER hit matching the regular expression CX_2_, CX_8_, ΨX_2_HX_3_, [H|C], where Ψ is a large, hydrophobic amino acid. Hits that did not match this expression and thus no longer have the structure necessary to bind DNA are considered degenerate, and are not identified as domains. HMMER hits below the corresponding bitscore thresholds but which matched this regular expression were retained in these proteins because C2H2-ZFs are known to occur in tandem, and therefore we are more confident about all C2H2-ZF domains which co-occur with at least one high scoring domain. All C2H2-ZF domains can be found in S5 Table.

Where possible, the longest protein splice form per gene containing all C2H2-ZF domains was selected to represent each gene. If no single protein isoform contained all domains present in the gene, a minimal set of proteins which together include all unique C2H2-ZF domains was selected to represent the gene.

### M2. Orthogroup Collection & Augmentation

A list of pairwise orthologs to *D. melanogaster* was downloaded from FlyBase and from the Andolfatto Lab build of *D. simulans* [102], and orthogroups were constructed from overlaps of these orthologs. Those orthogroups containing at least one *D. melanogaster* poly-ZF gene were selected. Of 13273 total original orthogroups, 272 had at least one *D. melanogaster* poly-ZF gene.

*D. melanogaster* poly-ZF orthogroups with sequences missing from one or more species were augmented according to the 15 insect whole genome alignment (WGA) from the UCSC Genome Browser [49]. A missing species is defined as any species not present in the orthogroup but present in the phylogenetic subtree rooted at the most recent common ancestor of those species that are present in the orthogroup. For each of the 52 orthogroups containing at least one missing species, known protein sequences were aligned to the UCSC 15-insect WGA using BLAT [104]. Where possible, sequence(s) from the missing species were extracted from the section of the alignment with the best hits and aligned back to their corresponding translated protein files using BLAT again. Gene IDs of proteins with BLAT hits with an e-value cutoff of 0.001 were extracted and, when they were not present in pseudogene lists, were added to the corresponding orthogroups. Through this process, 13 of the orthogroups with missing species were augmented with at least one new gene.

### M3. Orthogroup Reconciliation

All 1-to-many (i.e., one gene from *D. melanogaster* but more than one gene from at least one other species) orthogroups were truncated such that only those species with a single gene in the original orthogroup were included in the new orthogroup. In this manner, our analysis was restricted to variation in 1-to-1 orthologs.

A gene tree was constructed from a multiple alignment for each many-to-many orthogroup using T-Coffee, version 10 [105]. Each of these gene trees was then reconciled with the phylogenetic species tree for the 12 *Drosophila* species using Notung, version 2.8 [106]. For each input pair of gene and species trees, the reconciled tree output by Notung is marked with the most parsimonious duplication and loss events along ancestral branches, such that branches of the gene tree now coincide with speciation events of the species tree. Each subtree of the reconciled Notung tree was considered separately as a new potential orthogroup.

Potential orthogroups that contained fewer or greater than one *D. melanogaster* gene were discarded. All remaining potential orthogroups were truncated as before where necessary, such that only genes that were found to be 1-to-1 with a single *D. melanogaster* gene were retained. Potential orthogroups containing sequences from at least two species were extracted as new 1-to-1 orthogroups. Six original orthogroups were reconciled using Notung in this manner. All augmented and reconciled orthogroups can be found in S6 Table.

### M4. Binding Landscapes Across Species

We initially obtained binding specificity motifs, represented as PWMs, for 62 *D. melanogaster* poly-ZF genes from the FlyFactorSurvey, JASPAR, and public Transfac databases. There are 96 binding specificity motifs for these 62 genes, as different isoforms or subsets of binding domains may correspond to distinct motifs (e.g., peb_Z1-3 and peb_Z5-7). For cases of duplicate binding motifs, we preferentially selected the PWM generated from SOLEXA sequencing over SANGER sequencing, and the longer PWM over the shorter. To exclude binding motifs that are non-specific, we discarded PWMs with fewer than six columns exhibiting information content (IC) > 0.5. To exclude binding motifs of low complexity (e.g. poly-A motifs), we discarded PWMs where > 80% of columns with IC > 0.5 correspond to the same consensus nucleotide, where consensus is defined as the most common nucleotide in a position, or ‘N’ in the case of a tie. Slight variations to these thresholds do not affect our findings. To exclude TFs which cannot be compared across species, we discarded binding motifs corresponding to TFs with 1-to-1 orthologs in fewer than two non-reference species. This filtering process resulted in 64 binding specificity motifs for 52 genes. These motifs were properly formatted for use by fimo with jaspar2meme, available from the MEME suite [68].

The 2000 basepair regions upstream of all genes in *D. melanogaster* and their alignments to orthologous regions across the other 11 fly species were obtained from the UCSC Genome Browser 15-fly promoter region alignments [49]. For each binding specificity motif, fimo was run on these aligned upstream regions from all 12 fly species to find all predicted TF binding site occurrences.

To obtain a set of high-confidence predicted binding sites in *D. melanogaster*, we required that each predicted binding site in *D melanogaster* be found within 25 basepairs in the UCSC genome alignments to binding sites in *D. sechellia, D. simulans, D. yakuba*, and *D. erecta*; this allows detection of conserved sites while allowing for slight variations in the genomes and/or slight error in the genome alignment [107, 108]. We note that restricting *D. melanogaster* binding sites to those found within 15 or 50 basepairs to binding sites in these other four species did not affect results nor significance. Considering alternate definitions of confident binding sites by restricting *D. melanogaster* binding sites to those found within 25 basepairs in only *D. sechellia*, only *D. sechellia* and *D. simulans*, or only *D. sechellia, D. simulans*, and *D. yakuba* also did not affect results nor significance (S6 Fig.).

For each PWM, the set of “bound” promoter regions, or those containing one or more high-confidence binding sites, was obtained in *D. melanogaster*. For each of these bound promoter regions, the orthologous promoter region in a non-reference species was also considered bound if it contained one or more binding sites within 25 basepairs of a high-confidence *D. melanogaster* binding site. For each PWM, we were thus able to determine the percent of bound promoter regions in *D. melanogaster* that were also bound across each other fly species. Similarly, each high-confidence binding site in *D. melanogaster* was considered conserved in another species if a binding site was found in that species within 25 basepairs of the *D. melanogaster* binding site. If another binding site was not found in that species within this window, the high-confidence *D. melanogaster* binding site was considered lost. The proportion of orthologous promoter regions bound and proportion of binding sites conserved were calculated for each binding motif in each species that contained a 1-to-1 ortholog of the corresponding TF (Fig. 6B-C).

## Supporting Information

S1 Fig Overview of *Drosophila* C2H2-ZFs. (A) Distribution of the lengths of all identified C2H2-ZF domains across all species with a sequence logo of domains of length 21 amino acids, the most common domain length, shown. (B) The distribution of number of domains per *array*; a single protein sequence may contain multiple arrays of domains. An array is defined as adjacent C2H2-ZF domains separated by up to 12 amino acids. (C) Distribution of linker region (i.e., amino acid regions between adjacent C2H2-ZF domains) lengths with a sequence logo of the most common 7 amino acids long linker shown.
(TIF)

S2 Fig Conservation of canonically linked C2H2-ZFs. Overall and by-species divergence of aligned, canonically linked domains. A domain is considered diverged if it differs from its corresponding aligned *D. melanogaster* domain in one or more of the four specificity-determining positions -1, 2, 3, or 6. Divergence is shown according to (A) size of tandem array in which the domain appears, (B) average length of the linker(s) bordering the domain, and (C) position (beginning, middle, or end) of the domain. A domain may only fall into one of these three position categories; paired domains are labeled as beginning and end with no middle.
(TIF)

S3 Fig Folded site frequency spectra per species for different residue types. For each species, we determine amino acid residue sites that diverged with respect to *D. melanogaster* and give the folded site frequency spectra of those sites in *D. melanogaster*. Specifically, we show the proportion of polymorphic sites, categorized by amino acid residue type, where a minor allele was present in 1 through 69 individuals from a population of 139 *D. melanogaster* flies [59]. Only sites that are polymorphic within this *D. melanogaster* population and also diverged in a given species with respect to *D. melanogaster* are considered. Due to the low number of sites where 7 through 69 individuals exhibit the minor allele, these sites are aggregated under the “7+” label in *x*-axis. The four amino acid residue types are DNA-contacting residues (blue), background residues outside of C2H2-ZF domains (black), non-helical, non-binding residues within C2H2-ZF domains (red), and linker regions between adjacent canonically-linked domains (gray). The blue and black proportions are shown as bars for visual effect; we see that the minor allele frequencies for DNA-contacting residues are heavily skewed toward 0 in comparison to those for background residues. Red and gray residue types are shown as diamonds.
(TIF)

S4 Fig PCCs between SVM and ML/RF predicted specificities. Distribution of the PCCs between the SVM predicted binding specificity (PWM) and the ML predicted (red) and RF predicted (blue) binding specificities for all aligned domains from all *Drosophila* fly species. Blue vertical lines at 0, 0.25, 0.5, and 0.75 show the thresholds used for selecting confident predictions.
(TIF)

S5 Fig PCCs between *D. melanogaster* and non-reference predicted specificities. For each divergent non-*melanogaster* domain, we compared its SVM predicted specificity to the predicted specificity of its orthologous aligned *D. melanogaster* domain by calculating a PCC at each position b1 through b4. (A) Distribution of divergent domains per species by minimum PCC at any one position b1 through b4 from the aligned *D. melanogaster* domain. This shows that most divergent domains had a corresponding divergent binding specificity from *D. melanogaster* in at least one predicted position. (B) Distribution of divergent domains per species by sum of PCCs across positions b1 through b4 from the aligned *D. melanogaster* domain. All domains with a sum of PCCs < 2.0 must have had a divergent binding specificity in more than one predicted position from *D. melanogaster*.
(TIF)

S6 Fig Conservation of predicted *D. melanogaster* binding sites across species at varying confidence thresholds. We label each binding site in *D. melanogaster* as confident if it is found to be conserved (Methods M4) in *D. sechellia* (column 1), both *D. sechellia* and *D. simulans* (column 2), or *D. sechellia, D. simulans*, and *D. yakuba* (column 3). For each PWM listed in Fig. 6A, we calculate the percent of *D. melanogaster* promoter regions containing a confident binding site for that PWM that also contain a binding site in each of the other species (row 1, as in Fig. 6B) as well as the percent of confident *D. melanogaster* binding sites that are conserved in each other species (row 2, as in Fig. 6C). Points from species used for determining confident binding sites in *D. melanogaster* are excluded.
(TIF)

S7 Fig Expression of conserved and diverged poly-ZFs by tissue. (A) Percent of conserved (red) and diverged (blue) poly-ZF genes present in each tissue. FlyAtlas reports each gene as present or absent in each tissue separately across four replicates based on raw expression values [78]; we consider a gene to be present in a tissue if it was marked as present across all four of these replicates. Genes were marked as present or absent in each tissue. (B) Ubiquity of conserved and diverged poly-ZF genes according to the number of distinct tissues within the groups adult, larval, germline, and other they are present (binary score) in. (C) Raw expression level of each conserved and diverged poly-ZF gene by tissue type as a function of ubiquity as described in part B, with regression lines overlaid.
(TIF)

S1 Table Divergent residue counts and significance values. Counts of divergent residues and significance between binding residues and background per non-reference species used for the three calculations of functional importance previously described: (Major Column 1) Conservation Across Clades, (Major Column 2) Evolutionary Rate, and (Major Column 3) Rapid Fixation. The first four subcolumns within each major column correspond to the number of divergent residues in specificity-conferring positions -1, 2, 3, and 6 in C2H2-ZF domains (-1, 2, 3, 6), background divergent residues outside of arrays of canonically linked domains (BG), residues outside of the alpha-helix within C2H2-ZF domains (C2H2), and linker regions between adjacent canonically linked domains (linker). The fifth subcolumn in each major column is the exact *p*-value comparing the -1, 2, 3, 6 residues to BG residues using a binomial test in major columns 1 and 3 and Wilcoxon test in major column 2. In Major Column 1, residues are only included from each species where there is a non-gapped residue of the same type (e.g. -1, 2, 3, 6, BG, C2H2, linker) in an ortholog of its partner species. In Major Column 2, only complete (i.e., an ortholog from all 12 fly species) columns are included, as measurements of evolutionary rate using less complete multiple alignments could not be compared. In Major Column 3, all divergent residues, regardless of alignment to any species apart from the reference *D. melanogaster* are included.
(XLS)

S2 Table Percent of correctly-predicted columns by consensus predictions. Percent of columns from SVM-predicted binding specificities (represented as PWMs) that are “correct” (have a PCC > 0, 0.25, 0.5, or 0.75) when compared to the corresponding experimentally-derived gold standard specificity columns (S1 Text). Rows correspond to these correctness thresholds. We also consider subsets of SVM-predicted columns that agree (have a PCC > 0, 0.25, 0.5, or 0.75) with the predictions of ML or RF. Table columns correspond to these subsets of SVM-predicted columns defined by these consensus cutoffs. (A) Percent of correctly-predicted columns as compared to experimentally-derived PWMs for 158 natural poly-ZF TFs from all species. (B) Percent of correctly-predicted SVM columns compared to the 60 experimentally-derived PWMs from fly.
(XLS)

S3 Table Binding specificity change from reference (as measured by PCC) for various confidence thresholds. Change in binding specificity for aligned domains between the reference *D. melanogaster* and non-reference fly species. Confidence is measured by comparing SVM predicted specificities to those produced by ML and RF methods using PCC. The domains included at each confidence threshold are those where the SVM predicted specificities were within a particular PCC cutoff when compared to either the ML or RF predicted specificities. (Row 1) Total number of aligned, ungapped domains across all 12 fly species. (Row 2) Total number of ungapped *D. melanogaster* domains that have 1-to-1 orthologs in at least 1 other fly and exhibit a divergent binding residue in at least 1 other fly. (Row 3) Total number of aligned orthologous domains across *D. melanogaster* and at least 1 other fly species where the Spearman correlations relating the phylogenetic distance to *D. melanogaster* for each non-reference fly domain to the change in predicted specificity from the aligned *D. melanogaster* domain (measured using PCC, where a *lower* PCC implies *greater* change) is < 0. (Row 4) Total number of aligned orthologous domains where the Spearman correlations, as in Row 3, are < −0.5.
(XLS)

S4 Table Enrichment of Gene Ontology terms by GO TermFinder. GO Term Enrichment for *Drosophila* conserved and diverged poly-ZF genes as generated by GO Term Finder (go.princeton.edu).
(XLS)

S5 Table C2H2-ZF domains by species. C2H2-ZF domains found using HMMER in each protein in each *Drosophila* species (Methods M1). Columns are as follows: *Drosophila* species, genome build, FlyBase protein ID, FlyBase gene ID, domain position within protein, total number of C2H2-ZF domains in protein, Pfam ID of HMM that matched this domain, *e*-value of HMM match as computed by HMMER, bit score of HMM match as computed by HMMER, index of domain position -1 within protein sequence, index of domain position 7 within protein sequence, protein subsequence of positions -1 through 7 of domain, presence of an alignment gap between the HMM and the protein sequence between positions -1 and 7 of the domain, index of the starting cysteine of the domain in the protein sequence, index of the ending cysteine or histidine of the domain in the protein sequence, and the protein subsequence containing the entire C2H2-ZF domain.
(XLS)

S6 Table Augmented and reconciled orthogroups. Augmented and reconciled groups of *Drosophila* 1-to-1 orthologs (Methods M2 and M3). Columns are as follows: OrthoDB7 group ID, FlyBase protein ID, FlyBase gene ID, *Drosophila* species, UniProt ID, genome build, gene location (i.e., chromosome or scaffold name), gene start position on chromosome or scaffold, gene end position on chromosome or scaffold, gene coding strand on chromosome or scaffold (“+” is the forward strand and “-” is the reverse strand).
(XLS)

S1 Text Methods for evaluation of consensus prediction accuracy. (TXT)
