# Supplementary figures and images for: Pervasive Variation of Transcription Factor Orthologs Contributes to Regulatory Network Evolution
Source: PLoS Genet. 2015 Mar 6;11(3):e1005011. doi: 10.1371/journal.pgen.1005011 (PMC4351887; doi:10.1371/journal.pgen.1005011)

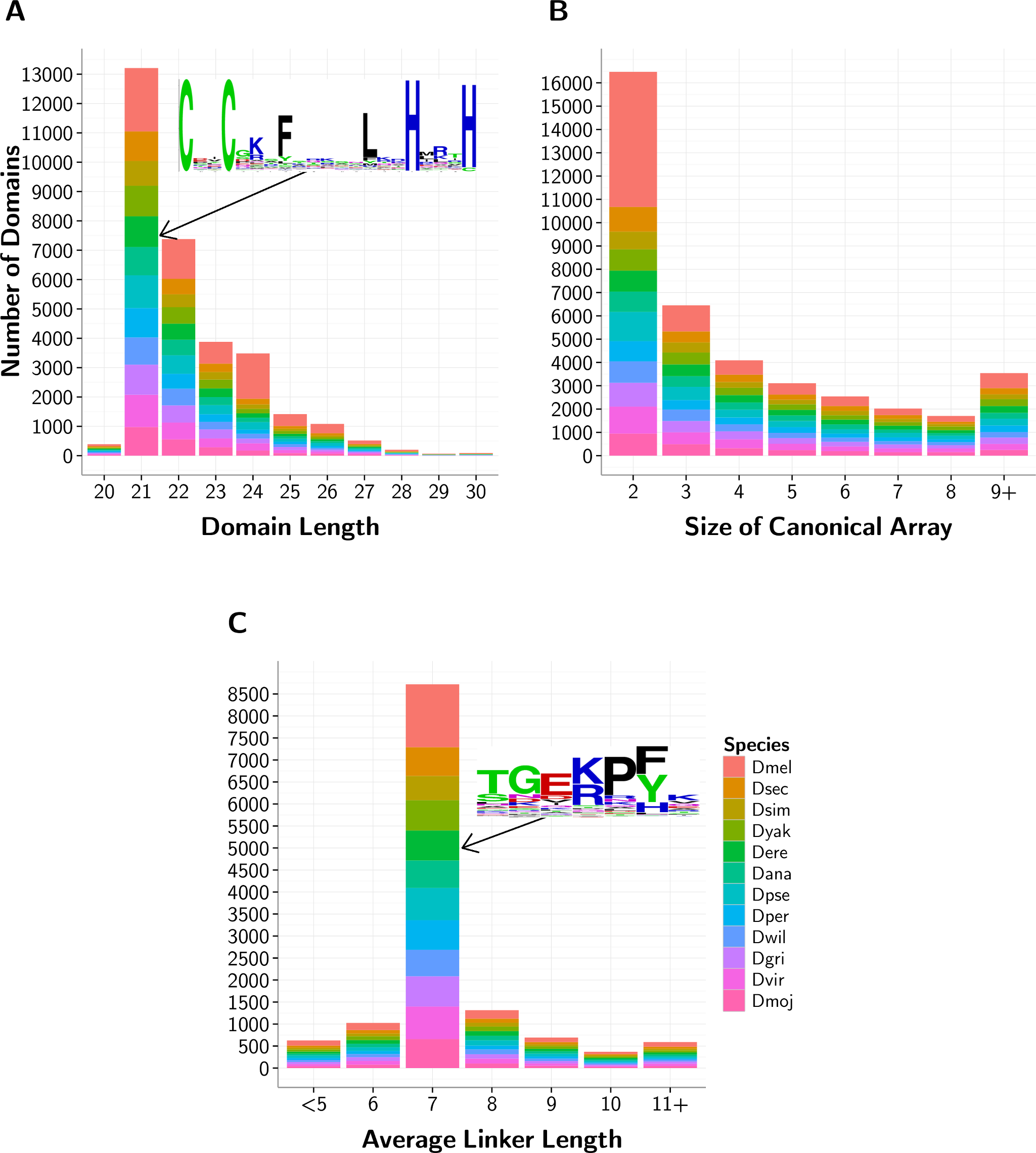

Supplement: S1 Fig — (A) Distribution of the lengths of all identified C2H2-ZF domains across all species with a sequence logo of domains of length 21 amino acids, the most common domain length, shown. (B) The distribution of number of domains per array; a single protein sequence may contain multiple arrays of domains. An array is defined as adjacent C2H2-ZF domains separated by up to 12 amino acids. (C) Distribution of linker region (i.e., amino acid regions between adjacent C2H2-ZF domains) lengths with a sequence logo of the most common 7 amino acids long linker shown. (TIF) [file pgen.1005011.s001.tif]

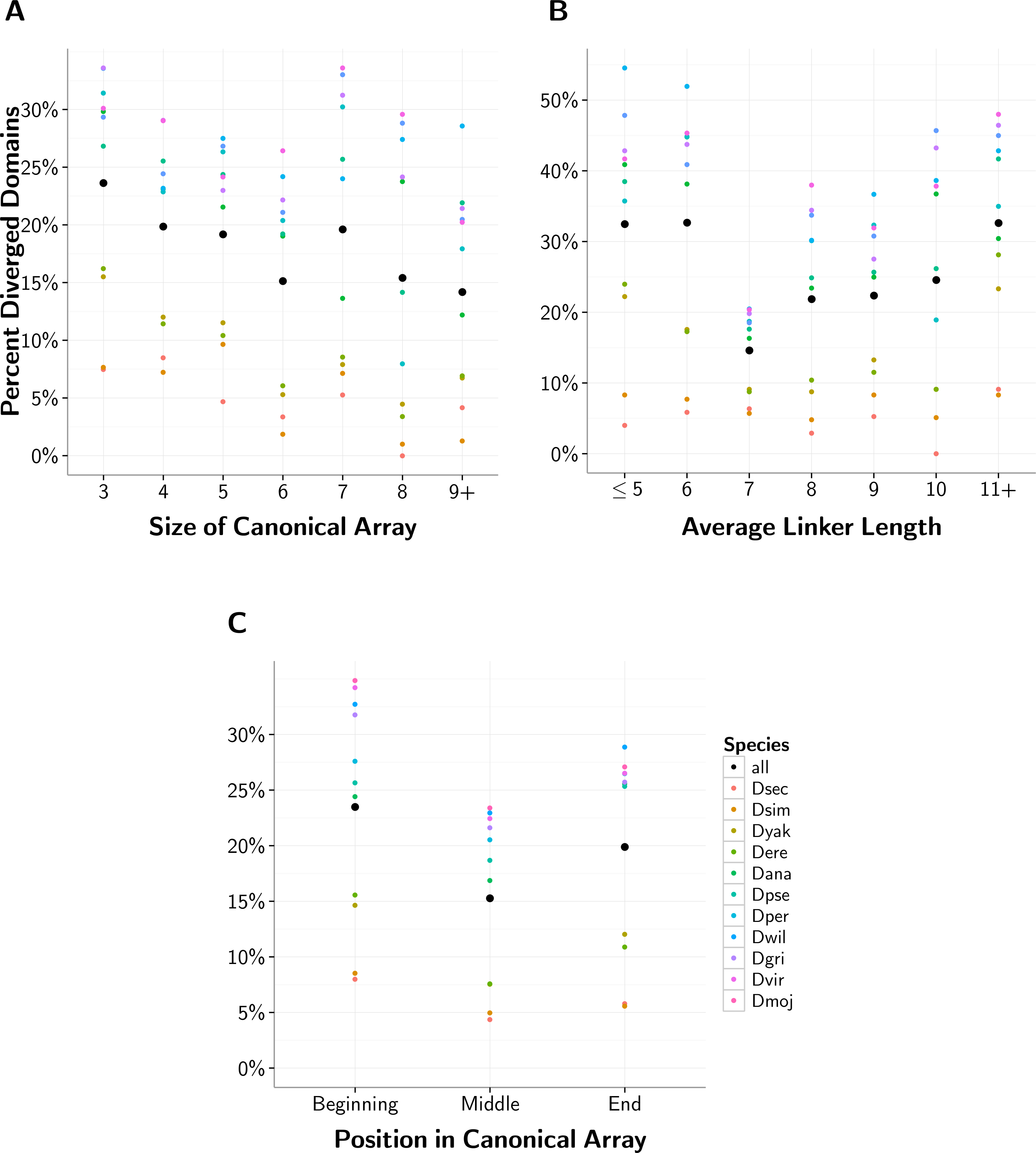

Supplement: S2 Fig — Overall and by-species divergence of aligned, canonically linked domains. A domain is considered diverged if it differs from its corresponding aligned D. melanogaster domain in one or more of the four specificity-determining positions -1, 2, 3, or 6. Divergence is shown according to (A) size of tandem array in which the domain appears, (B) average length of the linker(s) bordering the domain, and (C) position (beginning, middle, or end) of the domain. A domain may only fall into one of these three position categories; paired domains are labeled as beginning and end with no middle. (TIF) [file pgen.1005011.s002.tif]

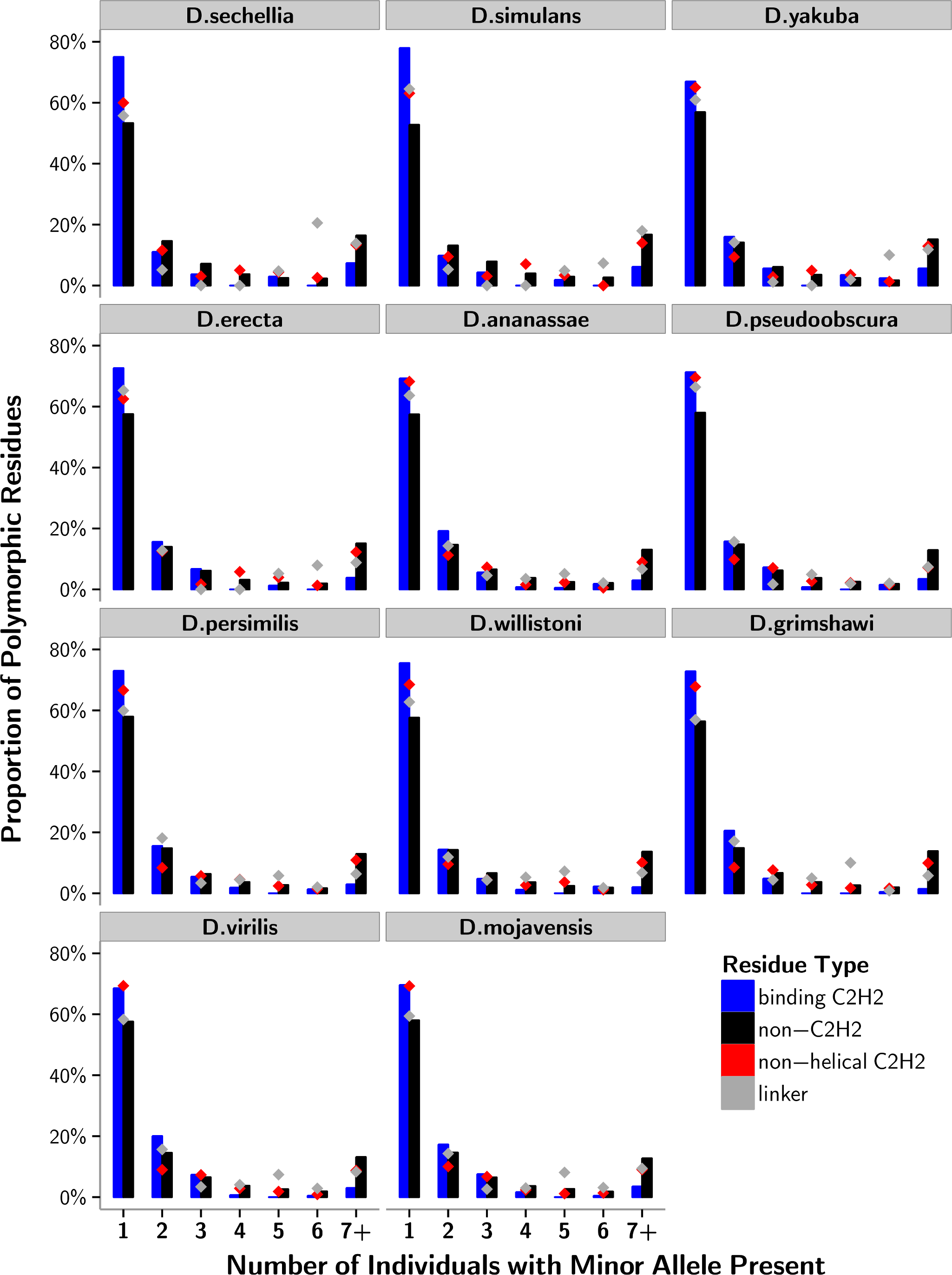

Supplement: S3 Fig — For each species, we determine amino acid residue sites that diverged with respect to D. melanogaster and give the folded site frequency spectra of those sites in D. melanogaster. Specifically, we show the proportion of polymorphic sites, categorized by amino acid residue type, where a minor allele was present in 1 through 69 individuals from a population of 139 D. melanogaster flies [59]. Only sites that are polymorphic within this D. melanogaster population and also diverged in a given species with respect to D. melanogaster are considered. Due to the low number of sites where 7 through 69 individuals exhibit the minor allele, these sites are aggregated under the “7+” label in x-axis. The four amino acid residue types are DNA-contacting residues (blue), background residues outside of C2H2-ZF domains (black), non-helical, non-binding residues within C2H2-ZF domains (red), and linker regions between adjacent canonically-linked domains (gray). The blue and black proportions are shown as bars for visual effect; we see that the minor allele frequencies for DNA-contacting residues are heavily skewed toward 0 in comparison to those for background residues. Red and gray residue types are shown as diamonds. (TIF) [file pgen.1005011.s003.tif]

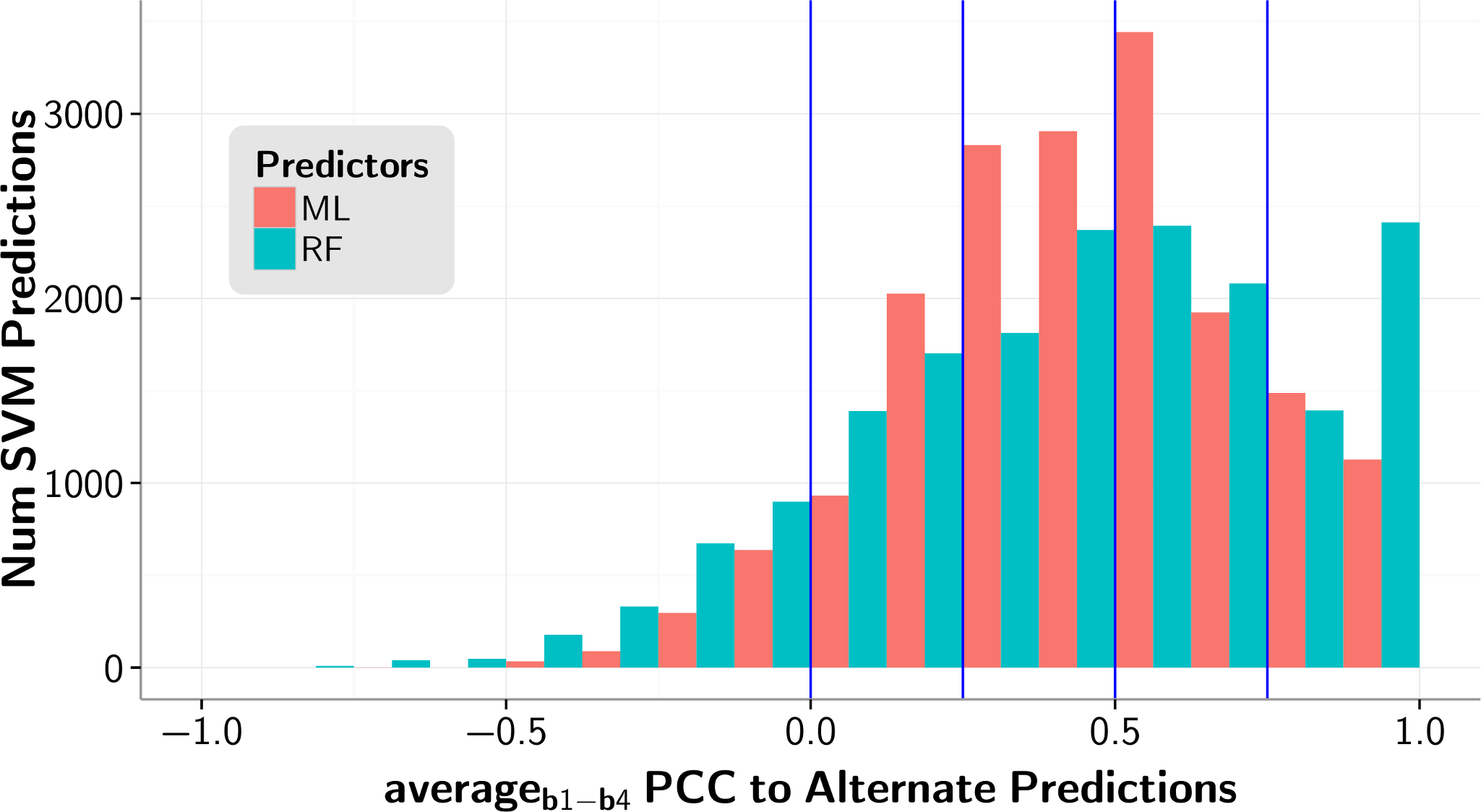

Supplement: S4 Fig — Distribution of the PCCs between the SVM predicted binding specificity (PWM) and the ML predicted (red) and RF predicted (blue) binding specificities for all aligned domains from all Drosophila fly species. Blue vertical lines at 0, 0.25, 0.5, and 0.75 show the thresholds used for selecting confident predictions. (TIF) [file pgen.1005011.s004.tif]

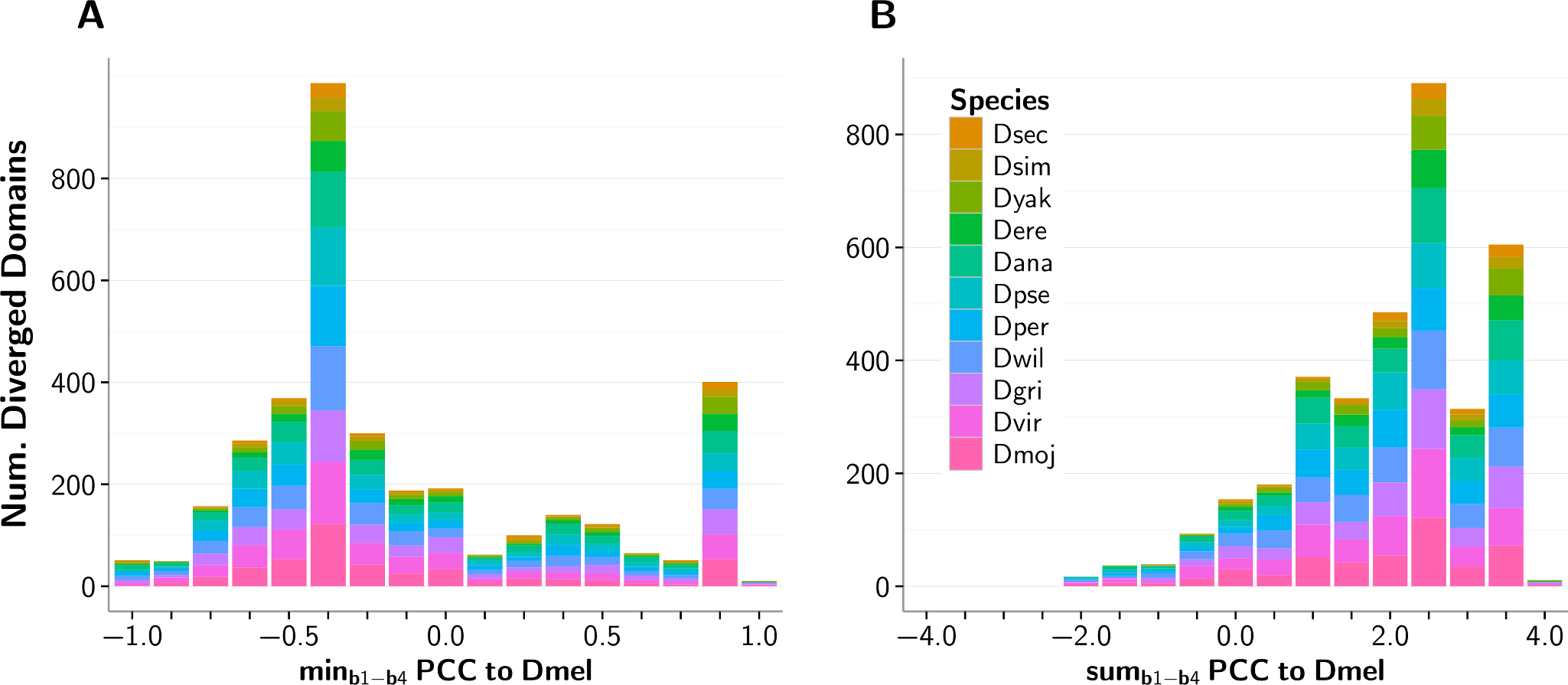

Supplement: S5 Fig — For each divergent non-melanogaster domain, we compared its SVM predicted specificity to the predicted specificity of its orthologous aligned D. melanogaster domain by calculating a PCC at each position b1 through b4. (A) Distribution of divergent domains per species by minimum PCC at any one position b1 through b4 from the aligned D. melanogaster domain. This shows that most divergent domains had a corresponding divergent binding specificity from D. melanogaster in at least one predicted position. (B) Distribution of divergent domains per species by sum of PCCs across positions b1 through b4 from the aligned D. melanogaster domain. All domains with a sum of PCCs < 2.0 must have had a divergent binding specificity in more than one predicted position from D. melanogaster. (TIF) [file pgen.1005011.s005.tif]

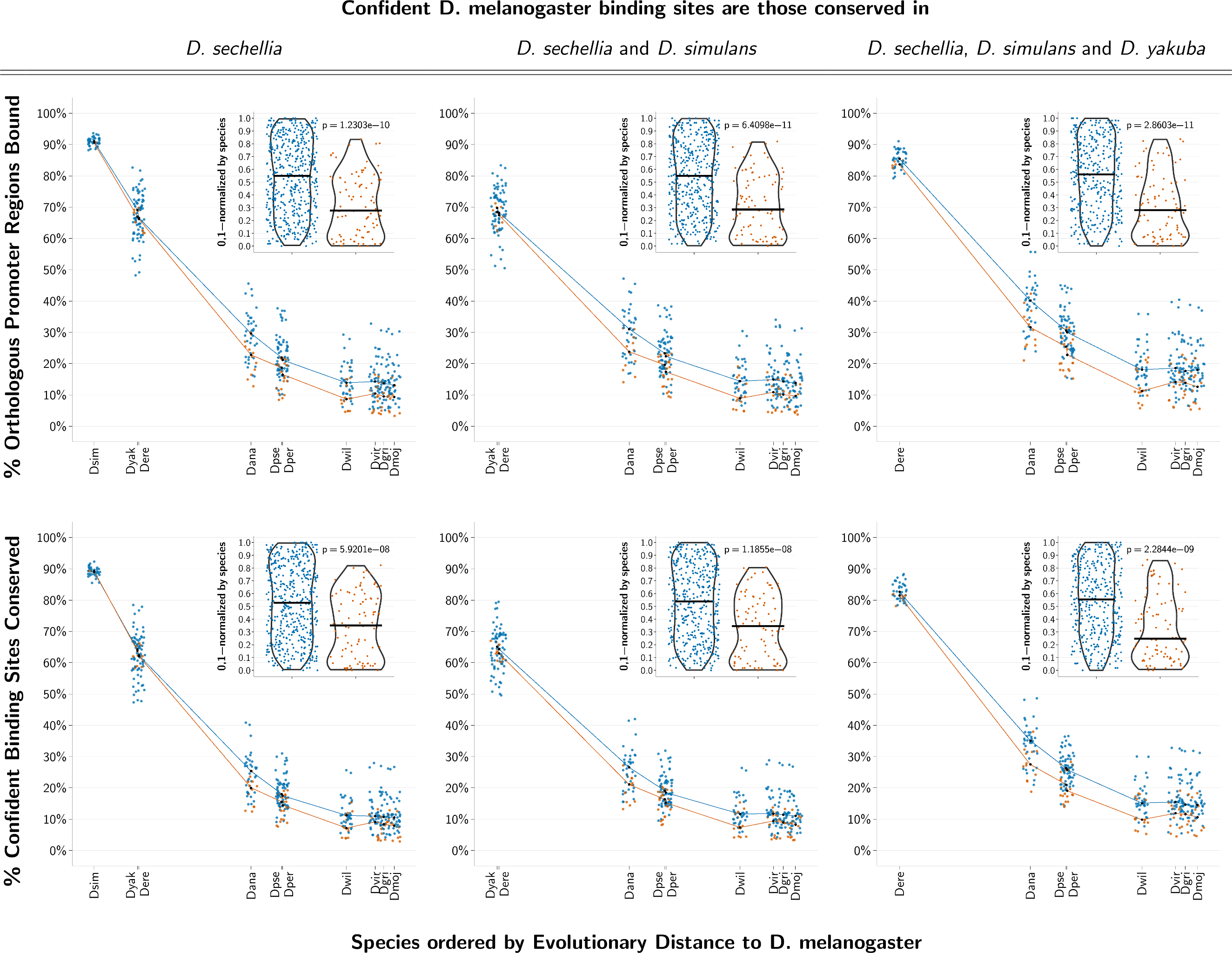

Supplement: S6 Fig — We label each binding site in D. melanogaster as confident if it is found to be conserved (Methods M4) in D. sechellia (column 1), both D. sechellia and D. simulans (column 2), or D. sechellia, D. simulans, and D. yakuba (column 3). For each PWM listed in Fig. 6A, we calculate the percent of D. melanogaster promoter regions containing a confident binding site for that PWM that also contain a binding site in each of the other species (row 1, as in Fig. 6B) as well as the percent of confident D. melanogaster binding sites that are conserved in each other species (row 2, as in Fig. 6C). Points from species used for determining confident binding sites in D. melanogaster are excluded. (TIF) [file pgen.1005011.s006.tif]

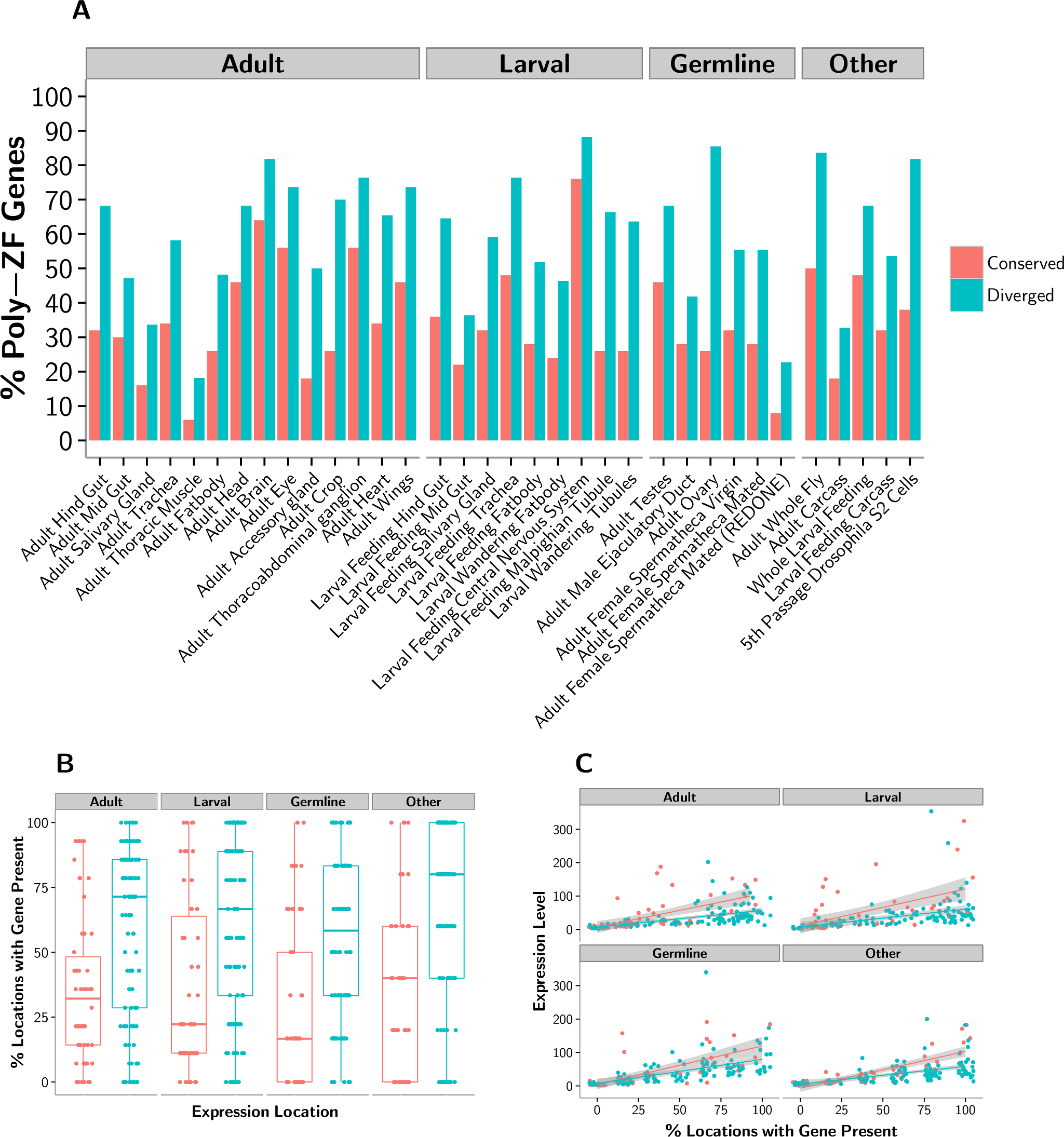

Supplement: S7 Fig — (A) Percent of conserved (red) and diverged (blue) poly-ZF genes present in each tissue. FlyAtlas reports each gene as present or absent in each tissue separately across four replicates based on raw expression values [78]; we consider a gene to be present in a tissue if it was marked as present across all four of these replicates. Genes were marked as present or absent in each tissue. (B) Ubiquity of conserved and diverged poly-ZF genes according to the number of distinct tissues within the groups adult, larval, germline, and other they are present (binary score) in. (C) Raw expression level of each conserved and diverged poly-ZF gene by tissue type as a function of ubiquity as described in part B, with regression lines overlaid. (TIF) [file pgen.1005011.s007.tif]
